# Risk and accuracy of outpatient-identified hypoxaemia for death among suspected child pneumonia cases in rural Bangladesh: a multifacility prospective cohort study

**DOI:** 10.1016/S2213-2600(23)00098-X

**Published:** 2023-09

**Authors:** Eric D McCollum, Salahuddin Ahmed, Arunangshu D Roy, ASMD Ashraful Islam, Holly B Schuh, Carina King, Shubhada Hooli, Mohammad Abdul Quaiyum, Amy Sarah Ginsburg, William Checkley, Abdullah H Baqui, Tim Colbourn

**Affiliations:** a Global Program in Pediatric Respiratory Sciences, Eudowood Division of Pediatric Respiratory Sciences, Department of Pediatrics, School of Medicine, Johns Hopkins University, Baltimore, MD, USA; b Department of International Health, School of Medicine, Johns Hopkins University, Baltimore, MD, USA; c Department of Epidemiology, School of Medicine, Johns Hopkins University, Baltimore, MD, USA; d Department of International Health, International Center for Maternal and Newborn Health, School of Medicine, Johns Hopkins University, Baltimore, MD, USA; e Bloomberg School of Public Health, and Division of Pulmonary and Critical Care, Department of Medicine, School of Medicine, Johns Hopkins University, Baltimore, MD, USA; f Center for Global Non-Communicable Disease Research and Training, School of Medicine, Johns Hopkins University, Baltimore, MD, USA; g Projahnmo Research Foundation, Dhaka, Bangladesh; h Department of Global Public Health, Karolinska Institutet, Stockholm, Sweden; i Section of Emergency Medicine, Department of Pediatrics, Baylor College of Medicine, Houston, TX, USA; j International Centre for Diarrhoeal Disease Research, Dhaka, Bangladesh; k Clinical Trials Center, University of Washington, Seattle, WA, USA; l Institute for Global Health, University College London, London, UK

## Abstract

**Background:**

Hypoxaemic pneumonia mortality risk in low-income and middle-income countries is high in children who have been hospitalised, but unknown among outpatient children. We sought to establish the outpatient burden, mortality risk, and prognostic accuracy of death from hypoxaemia in children with suspected pneumonia in Bangladesh.

**Methods:**

We conducted a prospective community-based cohort study encompassing three upazila (subdistrict) health complex catchment areas in Sylhet, Bangladesh. Children aged 3–35 months participating in a community surveillance programme and presenting to one of three upazila health complex Integrated Management of Childhood Illness (IMCI) outpatient clinics with an acute illness and signs of difficult breathing (defined as suspected pneumonia) were enrolled in the study; because lower respiratory tract infection mortality mainly occurs in children younger than 1 year, the primary study population comprised children aged 3–11 months. Study physicians recorded WHO IMCI pneumonia guideline clinical signs and peripheral arterial oxyhaemoglobin saturations (SpO_2_) in room air. They treated children with pneumonia with antibiotics (oral amoxicillin [40 mg/kg per dose twice per day for 5–7 days, as per local practice]), and recommended oxygen, parenteral antibiotics, and hospitalisation for those with an SpO_2_ of less than 90%, WHO IMCI danger signs, or severe malnutrition. Community health workers documented the children's vital status and the date of any vital status changes during routine household surveillance (one visit to each household every 2 months). The primary outcome was death at 2 weeks after enrolment in children aged 3–11 months (primary study population) and 12–35 months (secondary study population). Primary analyses included estimating the outpatient prevalence, mortality risk, and prognostic accuracy of hypoxaemia for death in children aged 3–11 months with suspected pneumonia. Risk ratios were produced by fitting a multivariable model that regressed predefined SpO_2_ ranges (<90%, 90–93%, and 94–100%) on the primary 2-week mortality outcome (binary outcome) using Poisson models with robust variance estimation. We established the prognostic accuracy of WHO IMCI guidelines for death with and without varying SpO_2_ thresholds.

**Findings:**

Participants were recruited between Sept 1, 2015, to Aug 31, 2017. During the study period, a total of 7440 children aged 3–35 months with the first suspected pneumonia episode were enrolled, of whom 3848 (54·3%) with an attempted pulse oximeter measurement and 2-week outcome were included in our primary study population of children aged 3–11-months. Among children aged 3–11 months, an SpO_2_ of less than 90% occurred in 102 (2·7%) of 3848 children, an SpO_2_ of 90–93% occurred in 306 (8·0%) children, a failed SpO_2_ measurement occurred in 67 (1·7%) children, and 24 (0·6%) children with suspected pneumonia died. Compared with an SpO_2_ of 94–100% (3373 [87·7%] of 3848), the adjusted risk ratio for death was 10·3 (95% CI 3·2–32·3; p<0·001) for an SpO_2_ of less than 90%, 4·3 (1·5–11·8; p=0·005) for an SpO_2_ of 90–93%, and 11·4 (3·1–41·4; p<0·001) for a failed measurement. When not considering pulse oximetry, of the children who died, WHO IMCI guidelines identified only 25·0% (95% CI 9·7–46·7; six of 24 children) as eligible for referral to hospital. For identifying deaths, in children with an SpO_2_ of less than 90% WHO IMCI guidelines had a 41·7% sensitivity (95% CI 22·1–63·4) and 89·7% specificity (88·7–90·7); for children with an SpO_2_ of less than 90% or measurement failure the guidelines had a 54·2% sensitivity (32·8–74·4) and 88·3% specificity (87·2–89·3); and for children with an SpO_2_ of less than 94% or measurement failure the guidelines had a 62·5% sensitivity (40·6–81·2) and 81·3% specificity (80·0–82·5).

**Interpretation:**

These findings support pulse oximeter use during the outpatient care of young children with suspected pneumonia in Bangladesh as well as the re-evaluation of the WHO IMCI currently recommended threshold of an SpO_2_ less than 90% for hospital referral.

**Funding:**

Fogarty International Center of the National Institutes of Health (K01TW009988), The Bill & Melinda Gates Foundation (OPP1084286 and OPP1117483), and GlaxoSmithKline (90063241).

**Research in context**

**Evidence before this study**

A comprehensive systematic review of 13 studies (two from South Asia) on hypoxaemic pneumonia in low-income and middle-income countries (LMICs) from Rahman and colleagues (2021) reported a prevalence of outpatient hypoxaemia of 23·1%. We did a supplemental search of Medline to identify literature on the prevalence of outpatient hypoxaemia from Oct 9, 2021, to June 12, 2022, including all languages, using these search terms: ((oximetry[MeSH Terms]) OR (hypoxia[MeSH Terms])) AND ((Child, Preschool[MeSH Terms]) OR (infant[MeSH Terms])). We searched Medline again for literature on hypoxaemia mortality with no language restrictions and without date restrictions using: (((oximetry[MeSH Terms]) OR (hypoxia[MeSH Terms])) AND ((Child, Preschool[MeSH Terms]) OR (infant[MeSH Terms]))) AND (mortality[MeSH Terms]). We found 220 publications on the prevalence of outpatient hypoxaemia and 512 on hypoxaemia mortality. Both searches were completed on June 12, 2022. We identified two additional studies not included in the systematic review: the EMPIC trial, which reported outpatient hypoxaemia prevalence and mortality from four LMICs (including Bangladesh), and one study from Malawi that reported outpatient hypoxaemia mortality (hypoxaemia prevalence estimates from this study were included in the systematic review). EMPIC was a cluster randomised controlled, open-label, non-inferiority trial conducted in Bangladesh, India, Ethiopia, and Malawi between 2016 and 2018 that compared a community-based amoxicillin treatment with treatment received in hospital in children aged 2–59 months with chest indrawing and without any WHO Integrated Management of Childhood Illnesses (IMCI) danger signs. Although the trial excluded participants with an peripheral arterial oxyhaemoglobin saturation (SpO_2_) of less than 90%, the prevalence and outcomes of these children were still reported, as were the prevalence and outcomes of children with an SpO_2_ of 90–92%. The reported outcomes were not stratified by study site. Among 582 children in Bangladesh with SpO_2_ measurements, the authors found six (1·0%) children with an SpO_2_ of less than 90% and 25 (4·3%) children with an SpO_2_ of 90–92%. Among the 732 children from India, only two (0·3%) children and 12 (1·6%) children had SpO_2_ measurements in these ranges. One of the ten deaths in EMPIC occurred in a child with an SpO_2_ of 90–92%, and only one death occurred in a child older than 12 months. The mortality risk of children aged 2–12 months in both study groups at all four sites was 0·4% (nine of 2179). Among all 2255 intervention group participants (children who received community-based amoxicillin treatment) aged 2–59 months with a recorded SpO_2_, 36 (1·6%) children had an SpO_2_ of less than 90% and all survived at 2 weeks. In the Malawi study of 675 children aged 0–59 months with WHO IMCI non-severe or severe pneumonia, children with an outpatient-measured SpO_2_ of less than 90% had an adjusted risk ratio for death of 9·3 (95% CI 2·1–40·4; p=0·003) compared with children with an SpO_2_ of 90–100%. For children with an outpatient-measured SpO_2_ of less than 93%, the adjusted risk ratio for death was 6·6 (1·5–29·4; p=0·012) compared with those with an SpO_2_ of 93–100%.

**Added value of this study**

To our knowledge, this study is the first to comprehensively evaluate the burden, mortality risk, and prognostic accuracy of hypoxaemia for identifying fatal cases among children with suspected pneumonia accessing outpatient care in South Asia. We report these endpoints for both the WHO-recommended threshold of an SpO_2_ of less than 90% and the alternative threshold of an SpO_2_ of less than 94%, with and without accounting for measurement failure.

**Implications of all the available evidence**

Our findings build upon the EMPIC results to further illuminate the added value of pulse oximeters for outpatient child pneumonia care in a South Asian context, and corroborate results from similarly designed studies conducted in sub-Saharan African settings. Altogether, the evidence should motivate LMICs to ensure children have routine access to pulse oximeters at outpatient clinics and for the re-evaluation of the WHO IMCI currently recommended threshold of an SpO_2_ of less than 90% for hospital referral.

## Introduction

According to 2017 estimates, lower respiratory infections (LRIs) killed approximately 800 000 children worldwide and were the leading infectious cause of paediatric deaths, ranking second overall.^1^, ^2^ Notably, nearly all LRI deaths occurred in low-income and middle-income countries (LMICs) and were concentrated among infants younger than 1 year; a third of global deaths were in South Asia.^1^ In South Asia, Bangladesh has a high incidence of pneumonia and approximately 20 000 children die from it annually.^1^, ^3^

Alveoli are air sacs in the lungs where respiratory gases are exchanged, and during severe LRIs they are prone to filling with inflammatory material or collapsing,^4^ especially in young children with small calibre airways and undeveloped collateral ventilation.^5^ The loss of alveoli participating in gas exchange can lower blood oxygen concentrations (hypoxaemia) because of a ventilation–perfusion mismatch.^6^ Among hospitalised children in LMICs with pneumonia, hypoxaemia increases mortality risk.^7^ In 2019, an estimated 7 million children in LMICs (95% CI 5–8 million) were hospitalised with hypoxaemic pneumonia.^8^

In Bangladesh, children commonly first encounter health-care providers outside of hospital, and outpatient health-care workers are recommended to follow WHO Integrated Management of Childhood Illnesses (IMCI) guidelines when caring for children with suspected pneumonia. WHO IMCI pneumonia recommendations help clinicians to decide between treatment with antibiotics in the outpatient setting or hospital referral.^9^ Pulse oximeter devices identify hypoxaemia by non-invasively measuring the peripheral arterial oxyhaemoglobin saturation (SpO_2_).^10^ The WHO IMCI guidelines recommend measuring SpO_2_ in children with suspected pneumonia because it can act as an indicator of severe disease warranting an escalation of care (a danger sign—ie, if found to be <90% the child is classified as having severe disease),^9^ but in practice oximeters are rarely used during outpatient care because of the restricted access to affordable, quality devices designed for children receiving health care in LMICs.^11^ In 2022, the Bangladesh Ministry of Health recommended introducing pulse oximetry into routine child health services, including clinics following WHO IMCI guidelines.^12^ However, the hypoxaemia burden among children at outpatient clinics in Bangladesh is poorly understood, as is the optimal SpO_2_ threshold for referral to hospital, and the mortality risk and prognostic accuracy of outpatient hypoxaemia identification in pneumonia cases. An improved understanding of these issues will allow the Bangladesh Ministry of Health to further refine pulse oximetry implementation approaches as well as aid international agencies and other LMIC health ministries to decide whether to invest in pulse oximeters for outpatient child pneumonia care.^12^, ^13^

To address these gaps, we conducted a prospective cohort study in rural Bangladesh at three outpatient IMCI clinics. Among young children aged 3–11 months (primary study population) and 12–35 months (secondary study population) with suspected pneumonia, we aimed to (1) estimate the outpatient hypoxaemia burden, (2) assess whether outpatient-identified hypoxaemia increases mortality risk at varying SpO_2_ thresholds, and (3) evaluate the prognostic accuracy of varying SpO_2_ thresholds for identifying fatalities.

## Methods

### Study design and participants

We implemented a prospective community-based cohort study nested within a pneumococcal conjugate vaccine effectiveness study.^14^ These studies were embedded within an ongoing population surveillance programme. The parent pneumococcal conjugate vaccine study was conducted between Jan 1, 2014, and June 23, 2018;^14^ this nested study was conducted from Sept 1, 2015, to Aug 31, 2017.

**Figure 1:**
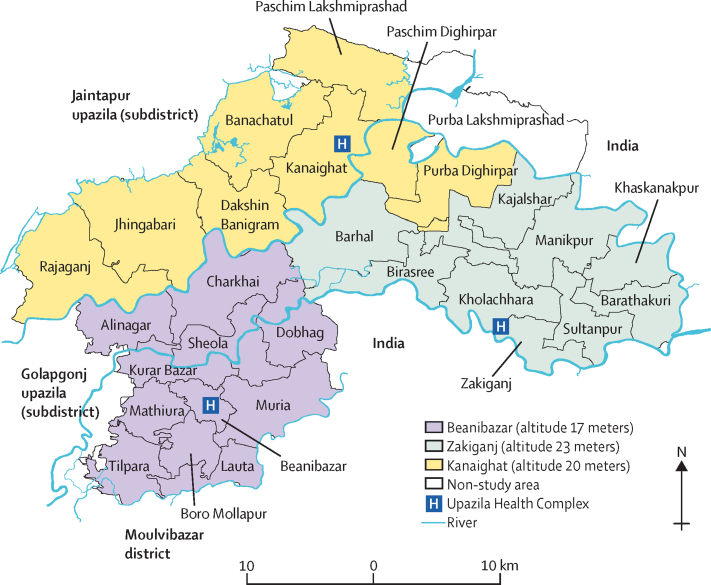
Projahnmo surveillance site

Johns Hopkins University, the Government of Bangladesh's Ministry of Health and Family Welfare, the International Centre for Diarrhoeal Disease Research of Bangladesh, Shimantik, the Child Health Research Foundation, and the Projahnmo Research Foundation comprise the research consortium Projahnmo. Since 2001, Projahnmo has conducted household surveillance in the Zakiganj upazila (an administrative region functioning as a subdistrict) in the Sylhet district of northeast Bangladesh. Surveillance expanded into the Kanaighat and Beanibazar upazilas for the pneumococcal conjugate vaccine study. Altogether, these three upazilas have a population of 800 000 and are at an altitude of 17–23 metres (figure 1).^14^, ^15^ Routine surveillance included household visits every 2 months by female community health workers, maintaining a census, and global positioning system mapping. Each community health worker surveyed 10 000 people; educated households for illness recognition (illness in general but with a focus on maternal and child health, including respiratory illness and pneumonia)**,** care-seeking, and pregnancy monitoring; and recorded vital status data (ie, whether the child was alive or dead). As a part of routine activities, trained data collectors conducted verbal autopsies for those who had died using a local instrument adapted from WHO tools. During the study period, approximately 8·1% of the surveillance population also participated in a separate study^14^ that evaluated weekly household respiratory surveillance by community health workers, including screening children for acute respiratory symptoms, counting respiratory rate, the use of pulse oximetry (in a subset), and recommending care at IMCI clinics when screening positive with fast breathing, WHO IMCI danger signs (appendix p 4) or an SpO_2_ of less than 90%.

Eligible participants for the current study were children aged 3–35 months—namely, because although the burden of hypoxaemia and hypoxaemia-associated mortality is probably highest in children younger than 12 months, the burden amongst those older than 12 months was not negligible in this setting and so we thought it was important to still evaluate—with suspected pneumonia, residing in the surveillance area, and seeking care (whether or not recommended by the community health worker) at participating clinics. Suspected pneumonia was adapted from WHO IMCI guidelines as an acute illness with observed signs of difficult breathing (appendix p 4).^9^ Study physicians evaluated children according to 2014 WHO IMCI guidelines,^9^ confirmed the presence of signs of difficult breathing (ie, suspected pneumonia), and collected SpO_2_, mid-upper arm circumference, weight, and temperature. Study physicians made clinical decisions considering the child's SpO_2_ measurement as recommended by WHO IMCI guidelines.^9^ Specifically, physicians recommended oxygen, parenteral antibiotics, and hospitalisation for children with a SpO_2_ of less than 90%, any WHO IMCI danger signs, or severe acute malnutrition. Study physicians counselled the caregivers of children eligible for hospitalisation on the importance of hospitalisation, parenteral antibiotics, and oxygen treatment. Participants not eligible for hospitalisation and those who declined hospitalisation were treated with oral amoxicillin (40 mg/kg per dose twice per day for 5–7 days, as per local practice). Participants were under routine surveillance, and as a part of this were visited at their household by community health workers once every 2 months. Written informed consent was obtained from caregivers by the study physicians. The Johns Hopkins Bloomberg School of Public Health (IRB 00005421) and School of Medicine (IRB00047406), Bangladesh Medical Research Council (BMRC/NREC/2013–2016/1008), and the Ethical Review Committee of the International Centre for Diarrhoeal Disease Research of Bangladesh (PR-13095) institutional review boards approved the study.

### Procedures and outcomes

From Sept 1, 2015, three Ministry of Health and Family Welfare IMCI clinics at upazila health complexes in Zakiganj, Kanaighat, and Beanibazar were enabled to identify suspected pneumonia in children aged 3–35 months. Projahnmo physicians were embedded within routine care services at the IMCI clinics to augment care and manage children according to 2014 WHO IMCI guidelines.^9^ Before study commencement, the Projahnmo physicians were trained on WHO IMCI guidelines and pulse oximetry. They measured SpO_2_ using a Masimo Rad-5 device with a LNCS Y-I wrap sensor (Masimo, Irvine, CA, USA) on the big toe of the children breathing in room air. Quarterly refresher trainings and monthly supportive supervision were provided. Pulse oximeter functionality was tested and verified once every 6 months using a ProSim SPOT Light SpO_2_ Pulse Oximeter Tester (FLUKE Biomedical, Everett, WA, USA).^16^ Upazila health complexes are subdistrict hospitals with paediatric services including an outpatient IMCI clinic and paediatric wards with 30 beds, with parenteral antibiotics, intravenous fluids, and a limited capacity to administer oxygen. As per standard practice in upazila health complexes, children requiring oxygen were referred to Sylhet M A G Osmani Medical College Hospital, a tertiary public hospital in Sylhet, because of an absence of specialist care and central oxygen supply. In coordination with each upazila health complex, the study provided medicines, portable oxygen, travel remuneration, and, if needed, direct transportation for participants requiring hospitalisation.

The primary outcome was death at 2 weeks after enrolment in children aged 3–11 months (primary study population) and 12–35 months (secondary study population), as recorded by community health workers conducting the household surveillance visits. The secondary outcome was 3-month mortality.

### Statistical analysis

**Figure 2:**
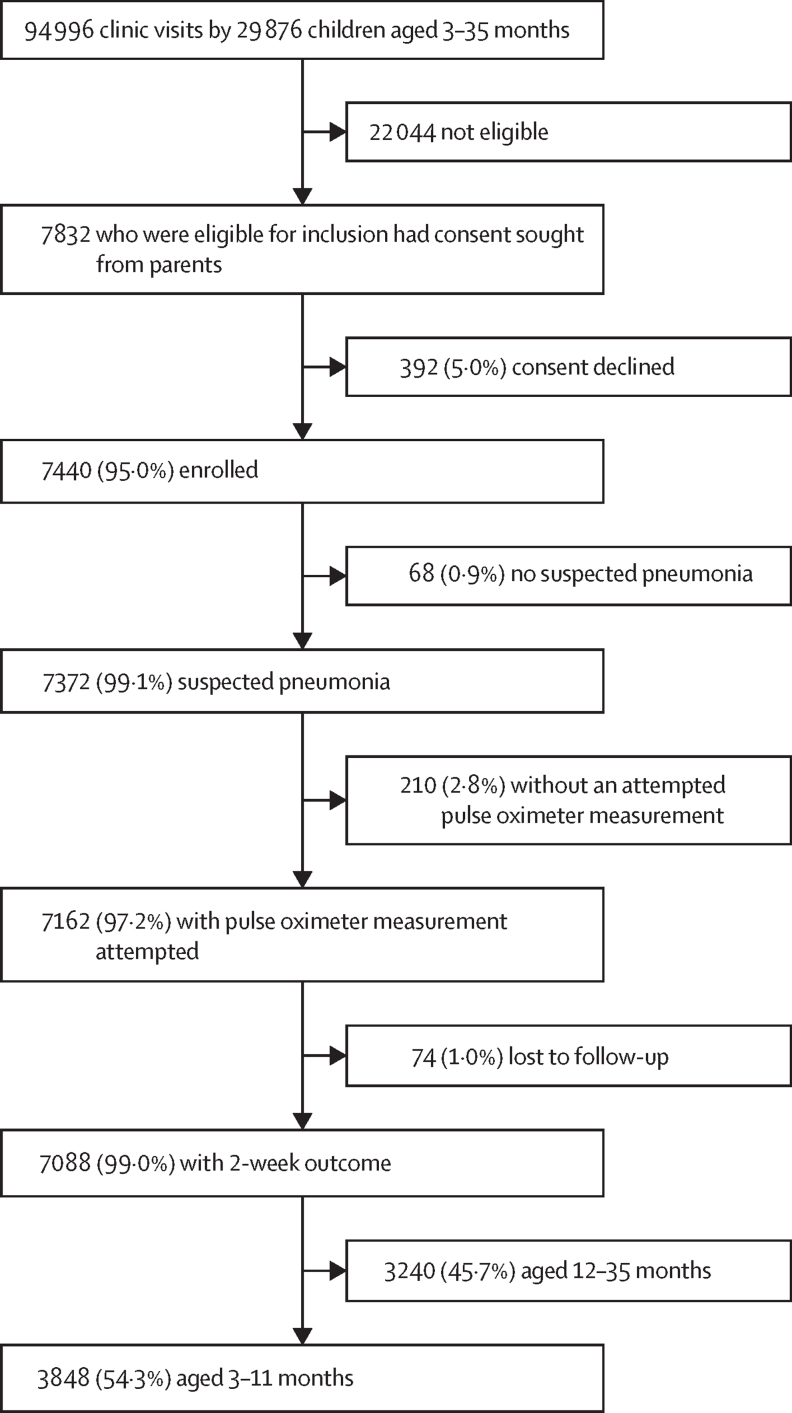
Study schema

Because LRI mortality mainly occurs in children younger than 1 year, our primary study population was children who were aged 3–11 months (established a priori) when the first episode of suspected pneumonia happened, recorded by the study and an attempted pulse oximeter measurement. Primary analyses (done in the primary study population) included estimating the outpatient prevalence, mortality risk at 2 weeks after enrolment, and the prognostic accuracy of hypoxaemia for death (2 weeks after enrolment) among children aged 3–11-months with suspected pneumonia. Children without an attempted pulse oximeter measurement were excluded (figure 2). We classified SpO_2_ as per data-driven thresholds derived from healthy Bangladeshi children aged 3–35 months (thresholds: <90%, 90–93%, and 94–100%) living in the same surveillance area with the same altitude.^15^ Community health worker surveillance data was linked to study physician clinical data using the child's permanent identification number available in both the surveillance and clinical visit data. At clinics, physicians entered their findings into a password-protected, encrypted computer. At the household level, community health workers recorded surveillance results on a paper-based register that was manually entered into a password-protected, encrypted computer by data entry clerks. To understand the added value of pulse oximeters during outpatient care, during analysis, children were reclassified as per WHO IMCI pneumonia categories assuming that oximetry and SpO_2_ measurements were unavailable.

Secondary analyses included estimating the prevalence, mortality risk (at 2 weeks post-enrolment), and the prognostic accuracy of hypoxaemia for death (2 weeks post-enrolment) in children aged 12–35 months, and also for death at 3 months post-enrolment in children aged 3–11 months and 12–35 months (appendix pp 5–13). Multiple post-hoc sensitivity analyses were also conducted to evaluate our primary findings, addressing any sources of potential bias or missing data (appendix pp 28–49). Post-hoc analyses of verbal autopsies were done using a symptom-based approach and the WHO's OpenVA platform (appendix pp 44–47).^17^

We did post-hoc analyses in the primary study population, including: decision curve analysis; applying the EMPIC trial eligiblity criteria^18^ to children aged 3–11 months with suspected pneumonia; difficulty breathing comparison among children aged 3–11 months; analysing children aged 3–11 months to assess whether including those referred to the clinics might have biased our results; evaluation of additional variables and 2-week mortality in models of children aged 3–11 months; weekly surveillance evaluation; 1-month mortality evaluation; verbal autopsy analysis; pneumococcal conjugate vaccine analysis; and probability of mortality by 1% SpO_2_ unit changes between 90 and 100%.

Mean (SD) and median (IQR) were used to summarise the continuous variables, and percentages were used for the categorical variables. Wilcoxon rank sum test, χ^2^, and Fisher's exact tests assessed crude associations between mortality and preselected exposure variables (age, sex, clinic location, WHO IMCI danger signs, severe acute malnutrition, SpO_2_, oxygen treatment, and hospitalisation). We produced risk ratios by fitting a multivariable model that regressed predefined SpO_2_ ranges (<90, 90–93, and 94–100) on the primary 2-week mortality outcome (binary outcome) using Poisson models with robust variance estimation.

Model specification included forward stepwise selection of statistically significant (p<0·05) variables in bivariable analysis, followed by comparing the model fit of the nested and unnested models by Bayesian information criterion (appendix pp 14–27). Bayesian information criterion allowed the prioritisation of model fit and parsimony because of the low number of observed deaths at 2 weeks (n=24). We tested model appropriateness and used non-parametric bootstrap estimation to perform internal validation of our final model (appendix p 21). We used the first episode of suspected pneumonia, because deaths among children having repeat visits (n=2593) were rare (n=2). We also evaluated the prognostic accuracy of WHO IMCI severe pneumonia criteria for identifying deaths at different SpO_2_ thresholds using sensitivity, specificity, positive and negative predictive values, positive and negative likelihood ratios, a likelihood-ratio test, area under the receiver operating characteristic curve, and the diagnostic odds ratio (OR),^19^ a ratio of the odds of WHO IMCI referral criteria (stridor at rest, convulsions, not feeding or drinking, vomiting everything, lethargy or coma, or severe acute malnutrition [weight-for-age z score of less than –3 or a mid-upper arm circumference of less than 11·5 cm, or both, for children older than 6 months]) positivity or hypoxaemia among those who died to the odds of WHO IMCI referral criteria positivity among those who survived. We estimated the burden of hospital referrals by clinic when applying each SpO_2_ approach. To generate these estimates we used observed study data to calculate the average number of participants with suspected pneumonia per day per clinic, and then multiplied this by the proportion of participants meeting the referral eligibility according to each SpO_2_ strategy. We then adjusted the resulting decimal fraction into a more interpretable nearest whole number ratio of referrals per number of days by taking the reciprocal of the decimal fraction and rounding it to the nearest whole number. Stata version 16.0 was used for analyses.

### Role of the funding source

The funders of the study had no role in study design, data collection, data analysis, data interpretation, or writing of the report.

## Results

During the study period of Sept 1, 2015, to Aug 31, 2017, a total of 7440 children who were aged 3–35 months, with their first suspected pneumonia episode identified during the study period, were enrolled (figure 2), and followed up for 3 months (mean 89·2 days [SD 7·5]). A pulse oximeter measurement was available for 7162 (97·2%) of 7372 children with suspected pneumonia, of whom 7088 (99·0%) of 7162 had a documented outcome. A total of 3848 (54·3%) of 7088 children were included in our primary study population of children aged 3–11 months and 3240 (45·7%) children aged 12–35 months were included in additional analysis.

All children aged 3–11 months suspected to have pneumonia were reclassified per WHO IMCI pneumonia categories assuming pulse oximetry was absent (table 1). Without considering pulse oximetry, a total of 313 (8·1%) of 3848 children had severe pneumonia, 2988 (77·7%) had non-severe pneumonia, and 547 (14·2%) had no pneumonia. The median age was 6 months (IQR 4–9) and 1595 (41·5%) children were girls. Fast breathing for age (2990 [77·7%]) and chest indrawing (1258 [32·7%]) were frequent. Although severe malnutrition was relatively frequent overall (307 [8·0%]), it was identified in nearly all severe pneumonia cases. Specifically, 307 (98·1%) of 313 severe pneumonia classifications were attributed to children with severe malnutrition. Although signs of respiratory distress (other than chest indrawing) are not included in the WHO IMCI guidelines, they were identified by study physicians in 384 (10·0%) children, including 327 (10·9%) of 2988 children with WHO IMCI non-severe pneumonia. In contrast, WHO IMCI danger signs were rare, with only eight (0·2%) children having any, all of whom had severe pneumonia. Among all children aged 3–11 months, 102 (2·7%) had an SpO_2_ of less than 90%, 306 (8·0%) had an SpO_2_ of 90–93%, and 67 (1·7%) had a failed SpO_2_ measurement (table 1). When also considering all children aged 3–11 months with suspected pneumonia, 10·6% (408/3848) had an SpO_2_ of less than 94%. 87 (2·9%) of 2988 children with WHO IMCI non-severe pneumonia had an SpO_2_ of less than 90% and 242 (8·1%) had an SpO_2_ of 90–93%, whereas three (0·5%) of 547 children classified as no pneumonia (per WHO IMCI guidelines) had an SpO_2_ of less than 90% and 29 (5·3%) children had an SpO_2_ of 90–93%. Although severe pneumonia requires hospitalisation, we found that most caregivers refused hospitalisation (253 [80·8%] of 313), despite recommendation from study physicians (611 children were recommended hospitalisation) and the study providing logistical support to facilitate it. In 3–11-month-old children, the primary outcome of death at 2 weeks after enrolment was low, at 0·6% (24 of 3848); at 3 months, case fatality doubled to 1·2% (47 of 3848). Most deaths at 2 weeks (16 [66·7%] of 24) and 3 months (28 [59·6%] of 47) had non-severe pneumonia when not considering SpO_2_ measurements for classifying WHO IMCI-defined pneumonia. There were two deaths during repeat clinic visits among 2593 children that visited the clinics more than one time. We conducted a post-hoc analysis applying EMPIC trial participant eligibility criteria to children aged 3–11 months in our study, and found that 0·5% (five of 1049) died 2 weeks after enrolment.

**Table 1:** Characteristics of outpatient children aged 3–11 months with suspected pneumonia in rural Bangladesh

		**No WHO IMCI pneumonia (n=547)**	**WHO IMCI non-severe pneumonia (n=2988)**	**WHO IMCI severe pneumonia (n=313)**	**Total (N=3848)**
Age, months		7 (5–9)	6 (4–8)	7 (4–9)	6 (4–9)
Sex					
	Girls	220 (40·2%)	1277 (42·7%)	98 (31·3%)	1595 (41·5%)
	Boys	327 (59·8%)	1711 (57·3%)	215 (68·7%)	2253 (58·5%)
Clinic					
	Beanibazar	356 (65·1%)	801 (26·8%)	78 (24·9%)	1150 (29·9%)
	Zakiganj	132 (24·1%)	861 (28·8%)	91 (29·1%)	1052 (27·3%)
	Kanaighat	246 (45·0%)	1326 (44·4%)	144 (46·0%)	1646 (42·8%)
Weight, kg		7·2 (1·1)	6·9 (1·1)	5·0 (0·9)	6·8 (1·2)
Weight-for-age z score					
	≥–2·0	271 (49·5%)	2519 (84·3%)	5 (1·6%)	3002 (78·0%)
	<–2·0 to ≥–3·0	100 (18·3%)	469 (15·7%)	5 (1·6%)	543 (14·1%)
	<–3·0	176 (32·2%)	..	303 (96·8%)	303 (7·9%)
Mid-upper arm circumference <11·5 cm		..	..	27 (8·6%)	27 (0·7%)
Severe acute malnutrition		..	..	307 (98·1%)	307 (8·0%)
Fever ≥101°F		61 (11·2%)	649 (21·7%)	62 (19·8%)	772 (20·1%)
Fast breathing for age		..	2728 (91·3%)	262 (83·7%)	2990 (77·7%)
Lower chest wall indrawing		..	1120 (37·5%)	138 (44·1%)	1258 (32·7%)
Respiratory distress		5 (0·9%)	327 (10·9%)	52 (16·6%)	384 (10·0%)
	Head nodding	2 (0·4%)	170 (5·7%)	31 (9·9%)	203 (5·3%)
	Nasal flaring	3 (0·5%)	173 (5·8%)	26 (8·3%)	202 (5·2%)
	Wheeze	3 (0·5%)	47 (1·6%)	5 (1·6%)	55 (1·4%)
	Grunting	..	20 (0·7%)	5 (1·6%)	25 (0·6%)
WHO IMCI danger signs		..	..	8 (2·6%)	8 (0·2%)
	Stridor	..	..	0	0
	Convulsion	..	..	5 (1·6%)	5 (0·1%)
	Not feeding	..	..	0	0
	Vomiting everything	..	..	1 (0·3%)	1 (<0·1%)
	Lethargy	..	..	2 (0·6%)	2 (0·1%)
SpO_2_ in room air*		97 (96–98)	97 (95–98)	97 (95–98)	97 (95–98)
	94–100%	508 (92·9%)	2608 (87·3%)	257 (82·1%)	3373 (87·7%)
	90–93%	29 (5·3%)	242 (8·1%)	35 (11·2%)	306 (8·0%)
	<90%	3 (0·5%)	87 (2·9%)	12 (3·8%)	102 (2·7%)
	Failed measurement	7 (1·3%)	51 (1·7%)	9 (2·9%)	67 (1·7%)
Hospitalisation		14 (2·6%)	284 (9·5%)	60 (19·2%)	358 (9·3%)
Oxygen		4 (0·7%)	127 (4·3%)	26 (8·3%)	157 (4·1%)
Mortality					
	2 weeks	2 (0·4%)	16 (0·5%)	6 (1·9%)	24 (0·6%)
	1 month	4 (0·7%)	17 (0·6%)	12 (3·8%)	33 (0·9%)
	3 months	5 (0·9%)	28 (0·9%)	14 (4·5%)	47 (1·2%)

Data shown as n (%), median (IQR), or mean (SD). Data were reclassified assuming that oxyhaemoglobin saturation data were unavailable. For definitions of WHO IMCI pneumonia, see the appendix (p 4). IMCI=Integrated Management of Childhood Illness. SpO_2_=peripheral arterial oxyhaemoglobin saturation.

* 540 measurements for no pneumonia category, 2937 measurements for non-severe pneumonia category, 304 measurements for severe pneumonia category, and 3781 measurements total.

In a bivariable analysis, IMCI clinic location (p=0·0331), severe acute malnutrition (p=0·0020), SpO_2_ categories (p<0·0001), hospitalisation (p<0·0001), and oxygen treatment (p<0·0001) were all associated with 2-week mortality children age 3–11-months (table 2). Death occurred by 2 weeks in 3·9% (four of 102) of children with an SpO_2_ of less than 90%, 1·6% (five of 306) with an SpO_2_ of 90–93%, 0·4% (12 of 3373) with an SpO_2_ 94–100%, and 4·5% (three of 67) with a failed SpO_2_ measurement. Compared with an SpO_2_ of 94–100%, an SpO_2_ of less than 90% when controlling for WHO IMCI severe pneumonia classification had an adjusted risk ratio of 10·3 (95% CI 3·2–32·3) for 2-week mortality (table 3), whereas the adjusted risk ratio of a failed SpO_2_ measurement was 11·4 (3·1–41·4), and an SpO_2_ of 90–93% was 4·3 (1·5–11·8). All sensitivity analyses and model selection outputs are in the appendix (pp 14–49), which include model testing for interactions and random effect of clinics. Adjusted risk ratios qualitatively similar to the above adjusted risk ratios were observed at 1 month (post-hoc analysis; appendix p 43) and 3 months (secondary analysis; appendix p 6) after enrolment, according to all additional post-hoc sensitivity analyses, including verbal autopsy analyses. Because the mortality events in children aged 12–35-months were rare (five [0·2%] of 3240), secondary analyses of this age range were limited to crude models only (appendix pp 7–11). A post-hoc analysis found a higher 2·3% (five of 220) mortality (2 weeks post-enrolment) among children aged 3-11 months with respiratory distress not reported by caregivers than the 0·5% (19 of 3628) mortality among those with respiratory distress reported by caregivers (p=0·001; appendix p 29). Another post-hoc analysis excluded 36 participants aged 3–11 months referred to the IMCI clinics, and found no qualitative difference from the results of primary analyses of all participants aged 3–11 months (appendix p 30). Lastly, we conducted a post-hoc analysis evaluating the effect of participants undergoing more intensive weekly surveillance on the primary analysis results. We found that although 13·2% (508 of 3848) of children aged 3–11 months with suspected pneumonia were visited weekly by community health workers, these participants had a similar distribution of hypoxaemia strata and mortality as participants not under weekly surveillance (appendix p 41).

**Table 2:** Bivariable analysis of associations with 2-week mortality in outpatient children aged 3–11 months with suspected pneumonia in rural Bangladesh

		**Alive (n=3824)**	**Dead (n=24)**	**p value**
Age, months		6·0 (4·0–9·0)	4·5 (3·5–7·5)	0·0709
Sex				
	Girls	1583 (41·4%)	12 (50·0%)	0·3937
	Boys	2241 (58·6%)	12 (50·0%)	..
Clinic				
	Beanibazar	1139 (29·8%)	11 (45·8%)	0·0331
	Zakiganj	1043 (27·3%)	9 (37·5%)	..
	Kanaighat	1642 (42·9%)	4 (16·7%)	..
Severe acute malnutrition*		301 (7·9%)	6 (25·0%)	0·0020
WHO IMCI danger signs†		8 (0·2%)	0 (0%)	0·8225
SpO_2_ in room air				
	94–100%	3361 (87·9%)	12 (50·0%)	<0·0001
	90–93%	301 (7·9%)	5 (20·8%)	..
	<90%	98 (2·6%)	4 (16·7%)	..
	Failed measurement	64 (1·7%)	3 (12·5%)	..
Hospitalisation		349 (9·1%)	9 (37·5%)	<0·0001
Oxygen		150 (3·9%)	7 (29·2%)	<0·0001

Data shown as n (%) or median (IQR). IMCI=Integrated Management of Childhood Illness. SpO_2_=peripheral arterial oxyhaemoglobin saturation. p values are shown for the overall categories.

* Weight-for-age z score of less than −3 or a mid-upper arm circumference of less than 11.5 cm or bilateral pedal oedema.

† Stridor at rest, convulsions, not feeding or drinking, vomiting everything, lethargy, or coma.

**Table 3:** Risk of 2-week mortality controlling for WHO IMCI referral criteria and SpO_2_ measurement in outpatient children aged 3–11 months with suspected pneumonia in rural Bangladesh

	Risk ratio*(95% CI)	**p value**	Adjusted risk ratio*(95% CI)	**p value**
**WHO IMCI and no SpO**_2_**measurement (n=3848)**				
WHO IMCI referral criteria†	3·8 (1·5–9·4)	0·0046	..	..
**WHO IMCI and SpO**_2_**continuous (n=3781)**				
WHO IMCI referral criteria†	3·8 (1·5–9·4)	0·0046	4·0 (1·5–10·7)	0·0054
SpO_2_	0·9 (0·8–0·9)	<0·0001	0·9 (0·8–0·9)	<0·0001
**WHO IMCI and SpO**_2_**strata (n=3848)**				
WHO IMCI referral criteria†	3·8 (1·5–9·4)	0·0046	3·1 (1·2–7·9)	0·0193
90–93%	4·6 (1·6–12·9)	0·0040	4·3 (1·5–11·8)	0·0048
<90%	11·0 (3·6–33·5)	<0·0001	10·3 (3·2–32·3)	0·0001
Failed SpO_2_measurement	12·6 (3·6–43·5)	0·0001	11·4 (3·1–41·4)	0·0002
**WHO IMCI and SpO**_2_**<90% or failed measurement attempt (n=3848)**				
WHO IMCI referral criteria†	3·8 (1·5–9·4)	0·0046	3·3 (1·2–8·7)	0·0163
<90% or failed SpO_2_measurement	9·0 (3·7–21·3)	<0·0001	8·2 (3·2–20·6)	<0·0001
**WHO IMCI and SpO**_2_**<94% or failed measurement attempt (n=3848)**				
WHO IMCI referral criteria†	3·8 (1·5–9·4)	0·0046	3·1 (1·2–7·9)	0·0159
<94% or failed SpO_2_measurement	7·1 (3·2–15·7)	<0·0001	6·6 (2·9–14·7)	<0·0001

WHO IMCI referral criteria refers to children with WHO IMCI severe pneumonia. IMCI=Integrated Management of Childhood Illness. SpO_2_=peripheral oxyhaemoglobin saturation.

* Risk ratio estimated by Poisson regression with robust variance estimation, and compared with an SpO_2_ of 94–100%.

† Any WHO IMCI referral criteria: stridor at rest, convulsions, not feeding or drinking, vomiting everything, lethargy or coma, or severe acute malnutrition (weight-for-age z score less than −3 or a mid-upper arm circumference less than 11·5 cm, or both [for children older than 6 months]).

**Figure 3:**
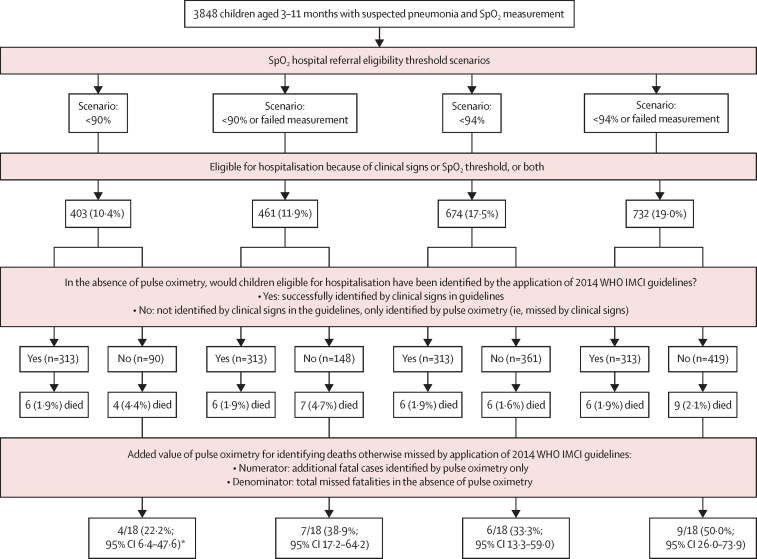
Added value of pulse oximetry to 2014 WHO IMCI guidelines for identifying deaths IMCI=Integrated Management of Childhood Illnesses. SpO_2_=peripheral arterial oxyhaemoglobin saturation. *Overall interpretation of less than 90% scenario: a total of 24 deaths occurred by 2 weeks, 18 of which were missed by clinical signs. Applying a less than 90% SpO_2_ threshold identified four (22·2%) more deaths than clinical signs alone. A total of ten deaths were identified assuming pulse oximetry was available and a less than 90% SpO_2_ threshold was applied.

Without pulse oximetry, the WHO IMCI classification of children aged 3–11 months did not identify 18 of 24 total fatalities at 2 weeks (75·0%; figure 3). Compared with a scenario without pulse oximeters, having oximetry available and using an SpO_2_ of less than 90% referral threshold identified 28·8% more children (90 of 313) for referral to hospital and 22·2% more deaths (four of 18 *vs* 0). Referring children with either an SpO_2_ of less than 90% or a failed measurement identified 47·3% (148 of 313) more children for referral and 38·9% (seven of 18 *vs* 0) more deaths than WHO IMCI classifications without considering pulse oximetry (ie, seven of 18 missed fatalities would be identified using an SpO_2_ of less than 90% or a failed measurement). Alternatively, referral for children with either an SpO_2_ of less than 94% or failed measurement identified 133·9% (419 of 313) more children for referral and 50·0% (nine of 18 *vs* 0) more deaths than WHO IMCI classification without pulse oximetry (ie, nine of 18 missed fatalities would be identified using an SpO_2_ of less than 94% or a failed measurement).

We compared the prognostic accuracy of the WHO IMCI pneumonia classification with and without information from pulse oximetry. Without pulse oximetry, the WHO IMCI severe pneumonia classification identified the lowest proportion of children aged 3–11-months for referral to hospital, at 8·1% (313 of 3848) and had the lowest sensitivity (25·0%, 95% CI 9·7–46·7) and specificity (92·0%; 91·1–92·8) for identifying deaths (table 4). With pulse oximeters and a less than 90% SpO_2_ threshold, the WHO IMCI classification identified 10·5% (403 of 3848) of children for referral and identified deaths with a 41·7% sensitivity (22·1–63·4) and 89·7% specificity (88·7–90·7). We examined two alternative approaches, where a combination of measurement failure with either an SpO_2_ of less than 90% or 94% prompts referral, and we found that both combinations outperformed WHO IMCI classification without pulse oximetry and WHO IMCI classification using an SpO_2_ of less than 90% or 94%. An SpO_2_ of less than 90% or measurement failure identified 12·0% (461 of 3848) of children for referral and had the highest diagnostic odds ratio for identifying deaths at 8·9 (95% CI 3·9–20·0). However, an SpO_2_ of less than 94% or measurement failure identified the highest proportion of children for referral at 19·0% (732 of 3848) and achieved a 62·5% sensitivity (40·6–81·2) and 81·3% specificity (80·0–82·5) for identifying deaths (table 4). Using these performance profiles, over the 2-year study period, WHO IMCI classification of pneumonia severity without pulse oximetry would be projected to refer about one child aged 3–11 months per clinic to the hospital every 7 days. If a threshold of an SpO_2_ of less than 90% is used, then one child per clinic would be referred every 5–6 days. Alternatively, an SpO_2_ of less than 90% or measurement failure would identify one child per clinic for referral every 5 days, whereas an SpO_2_ of less than 94% or measurement failure would identify one child per clinic every 3 days.

**Table 4:** Accuracy of WHO IMCI guidelines for identifying 2-week mortality with and without pulse oximetry and at varying SpO_2_ thresholds for outpatient children aged 3–11 months in Bangladesh

	**Referral eligibility prevalence, % (n/N)**	**Sensitivity for identifying deaths (95% CI)**	**Specificity for identifying deaths (95% CI)**	**Positive predictive value for mortality (95% CI)**	**Negative predictive value for mortality (95% CI)**	**Likelihood ratio positive (95% CI)**	**Likelihood ratio negative (95% CI)**	**AUC**	**Diagnostic odds ratio for identifying deaths (95% CI)**	Likelihood ratio test*
WHO IMCI without pulse oximetry	8·1% (313/3848)	25·0% (9·7–46·7)	92·0% (91·1–92·8)	1·9% (0·7–4·1)	99·5% (99·2–99·7)	3·1 (1·5–6·2)	0·8 (0·6–1·0)	0·58 (0·49–0·67)	3·8 (1·5–9·6)	Ref
WHO IMCI with SpO_2_ <90%	10·5% (403/3848)	41·7% (22·1–63·4)	89·7% (88·7–90·7)	2·4% (1·2–4·5)	99·6% (99·3–99·8)	4·0 (2·5–6·5)	0·6 (0·4–0·9)	0·65 (0·55–0·75)	6·2 (2·7–14·1)	7·9
WHO IMCI with SpO_2_ <90% or failed measurement	12·0% (461/3848)	54·2% (32·8–74·4)	88·3% (87·2–89·3)	2·8% (1·5–4·7)	99·7% (99·4–99·8)	4·6 (3·1–6·7)	0·5 (0·3–0·8)	0·71 (0·61–0·81)	8·9 (3·9–20·0)	15·2
WHO IMCI with SpO_2_ <94%	17·5% (674/3848)	33·3% (15·6–55·3)	84·9% (83·8–86·1)	1·3% (0·5–2·6)	99·5% (99·2–99·7)	2·2 (1·2–3·9)	0·7 (0·5–1·0)	0·59 (0·49–0·68)	2·8 (1·2–6·6)	11·0
WHO IMCI with SpO_2_ <94% or failed measurement	19·0% (732/3848)	62·5% (40·6–81·2)	81·3% (80·0–82·5)	2·0% (1·1–3·3)	99·7% (99·5–99·9)	3·3 (2·4–4·5)	0·4 (0·2–0·7)	0·71 (0·62–0·81)	7·2 (3·1–16·5)	18·6

WHO IMCI guidelines refer to children categorised into having WHO IMCI severe pneumonia. AUC=area under the receiver operating characteristic curve. IMCI=Integrated Management of Childhood Illnesses. SpO_2_=peripheral oxyhaemoglobin saturation.

* χ^2^ test statistic for the likelihood ratio test comparing the reduced model (WHO IMCI without pulse oximetry) and the full model (WHO IMCI with pulse oximetry [SpO_2_ threshold specified by table row]). p values for all likelihood ratio test comparisons <0·01.

## Discussion

We conducted a multifacility, prospective, observational study in rural Bangladesh to evaluate the potential effect of pulse oximetry implementation on the WHO IMCI-based outpatient care of 3848 young children with suspected pneumonia. There are four key findings. First, although hypoxaemia prevalence and mortality were low in this outpatient population, applying WHO IMCI guidelines without pulse oximetry identified few children who died as being high risk. None of the four deaths with an SpO_2_ of less than 90% at presentation and just 25% of all deaths (six of 24) were eligible for referral based on WHO IMCI classification without considering pulse oximetry. Second, when compared with clinical signs, an SpO_2_ of less than 90% only modestly improved WHO IMCI classification performance for identifying deaths. This implies that using a threshold of an SpO_2_ of less than 90% for hospitalisation might have a lower than desired effect on mortality reductions in Bangladesh and similar LMICs. Third, however, using a strategy that recognised an SpO_2_ of less than 94% or failed measurement for referral identified children at high risk of mortality and had the highest sensitivity of the four examined strategies for identifying deaths. Although requiring more evaluation, implementation of this strategy appears feasible, because it would have referred two infants per week from each of the three participating clinics. Lastly, we observed that a high proportion of caregivers of children eligible for hospitalisation refused hospitalisation, despite counselling and the availability of logistical support services, including transportation. A similarly high 83% hospitalisation refusal rate has been previously reported among caregivers of septic infants in Bangladesh, and attributed to economic barriers, household factors, and previous experience of poor quality of care at hospitals.^20^ This finding is concerning, and further research is needed to understand its implication on pneumonia mortality reduction strategies (including oxygen treatment) that can only be delivered when patients are hospitalised, and to examine why there is a low uptake for hospitalisation and how to mitigate this.

When using an SpO_2_ threshold of less than 90%, only 2·7% (102 of 3848) of children with suspected pneumonia were hypoxaemic in this study. When an SpO_2_ of less than 94% was used, then hypoxaemia prevalence increased to 10·6% (408 of 3848). Regardless of the hypoxaemia threshold, our estimates differ from the 23·1% outpatient prevalence of hypoxaemia found in a 2022 meta-analysis.^8^ Of the 13 studies contributing outpatient data in the meta-analysis, two were from the South Asian region and six enrolled individuals with severe disease only. Direct comparisons with the two included South Asian studies are constrained because they included different ages, disease severities, altitudes, hypoxaemia thresholds, and time periods. Altogether, these likely limit the generalisability of the meta-analysis to the South Asian outpatient context.

A multicountry, cluster, randomised controlled, open-label, non-inferiority trial published in 2022 called EMPIC was not included in the meta-analysis, but its findings are more similar to this study, and it included Bangladesh.^18^ The trial evaluated community-based amoxicillin treatment of children aged 2–59 months in Bangladesh, India, Ethiopia, and Malawi between 2016 and 2018. A total of 3897 participants with chest indrawing, an SpO_2_ of 90% or more, and without any WHO IMCI danger signs were analysed. 25 (4·3%) of 582 EMPIC participants in Bangladesh had an SpO_2_ of 90–92%, lower than the 306 (8·0%) of 3848 participants who had an SpO_2_ of 90–93% in this study. Overall, nine of ten deaths in the EMPIC study occurred in children aged 2–11 months with a mortality of 0·4% (nine of 2179) in this age range, and one child who died had an SpO_2_ of 90–92%. The authors did not report in which country the deaths occurred. To facilitate comparison, post-hoc we re-analysed our sample (appendix p 29) using EMPIC eligibility criteria and found a similar 0·5% (five of 1049) mortality rate among children aged 3–11 months in our study.

The contrast in illness severity between studies might be explained by the outpatient care pathway in Bangladesh as well as differing screening approaches for eligibility. Notably, EMPIC reported that as many as 40% of caregivers in Bangladesh first access care at health clinics similar to the upazila health clinics in our study, which suggests our study might be more representative of the entire spectrum of paediatric outpatients in Bangladesh. It is possible that primary care patients with more advanced illness bypassed the EMPIC community clinics altogether or eligibility criteria excluded them. As stipulated by WHO IMCI guidelines,^9^ the EMPIC study had caregivers subjectively report respiratory complaints before the study staff screened children for inclusion. Given the known limited caregiver knowledge on pneumonia symptoms throughout rural Bangladesh, we did not rely on caregiver reporting but systematically screened for respiratory signs regardless of elicited symptoms.^21^ Our post-hoc sensitivity analysis confirms that patients in our study with respiratory distress not reported by caregivers had a higher mortality than those recognised and reported by caregivers (appendix p 29; 2·2% *vs* 0·5%, p=0·001). Both issues probably contribute to the EMPIC trial's milder disease profile in its participants.

Although an elevated mortality risk measured by SpO_2_ ranges more than 90% might be novel in South Asia, similarly elevated mortality risks in children in both outpatient and hospital settings have been reported in Malawi.^22^, ^23^, ^24^ In Malawi a 6·6 adjusted risk ratio for death (95% CI 1·5–29·4, p=0·012) was found for an outpatient-measured SpO_2_ threshold of less than 93%, compared with an SpO_2_ of 93–100%, among 695 children aged 0–59 months with WHO IMCI non-severe or severe pneumonia.^22^ Among 14 665 hospitalised children aged 2–59 months with an SpO_2_ of 90–92%, compared with an SpO_2_ of 93–100%, a 1·54 adjusted odds ratio for death (1·05–2·28) was observed; and among 1088 hospitalised children aged 0–2 months with an SpO_2_ of 90–92% compared with an SpO_2_ of 93–100%, a 4·3 adjusted odds ratio (1·7–11·1) for death was found.^23^, ^24^

Although children with an SpO_2_ of 90–93% have a higher mortality risk, whether they benefit from hospitalisation and oxygen is uncertain. One multifacility randomised trial from the UK of 615 hospitalised infants aged 6 weeks to 12 months with physician-diagnosed bronchiolitis showed no difference in time to cough resolution with oxygen treatment when comparing an SpO_2_ threshold of less than 94% with a threshold of less than 90% (where infants with an SpO_2_ of 90–93% did not receive oxygen).^25^ No infants died in the untreated SpO_2_ 90–93% group and non-fatal adverse events were infrequent. The authors concluded that infants with bronchiolitis and an SpO_2_ of 90–93% could be safely and effectively treated without oxygen.

However, mortality among children with an SpO_2_ of 90–93% in LMICs is markedly higher compared with higher income countries such as the UK. More than double the percentage of children in Bangladesh (approximately 40%) are estimated to be anaemic compared with the UK.^26^ Aerobic cellular metabolism requires oxygen, and the delivery of oxygen in the blood primarily relies on haemoglobin.^27^ Therefore, on average, at any given SpO_2_ concentration, children in Bangladesh have comparatively less oxygen circulating in the body. Additionally, SpO_2_ measurements among individuals with darker skin pigmentation, compared with lighter pigmentation, might be systematically higher than actual arterial oxyhaemoglobin saturations, possibly from pigment characteristics influencing optical factors used by pulse oximeters to generate measurements.^28^ Given the demographics of both countries (Bangladesh and the UK), this suggests that an unknown percentage of SpO_2_ measurements in hypoxaemic Bangladeshi children might be falsely negative, an issue termed occult hypoxaemia.^28^ Additionally noteworthy is that Bangladesh has some of the highest amounts of air pollution in the world, as well as elevated childhood malnutrition rates.^29^, ^30^ In this study we observed that nearly all children with severe pneumonia had disease complicated by severe malnutrition. It is likely that in Bangladeshi children, their underlying lung health and pulmonary reserve, compared with children in the UK, is impaired in part because of these issues, increasing the vulnerability of children in Bangladesh with an SpO_2_ of 90–93%. Lastly, the health systems in both countries differ such that UK children generally have earlier access to care and follow-up and better access to diagnostic testing, all of which might facilitate the more timely detection and treatment of children with disease progression.

We also found failed SpO_2_ measurements to be important, independently conveying a 10-times higher adjusted risk ratio for death than an SpO_2_ of 94–100%. This finding is also consistent with previous observations from Malawi.^22^, ^23^ The outpatient Malawi study reported a 10·8 adjusted risk ratio (95% CI 1·02–113·1) for death with failed SpO_2_ measurement compared with an SpO_2_ of 90–100%.^22^ Another analysis of 1879 hospitalised Malawian infants younger than 2 months found an 18·1 adjusted odds ratio (7·5–42·8) for death with failed SpO_2_ compared with an SpO_2_ of 93–100%.^23^ User error, poor probe fit, movement artifact, or device malfunction might account for some of these failed measurements, but this effect is likely to be small, as has been previously observed in pilot implementation research from Bangladesh and other settings.^31^, ^32^, ^33^ Another key explanation for measurement failure could be inadequate peripheral perfusion from unrecognised shock. Current WHO IMCI danger signs do not have any specific clinical sign for impaired perfusion,^9^ which is both a recognised clinical sign of shock and can lead to pulse oximeter measurement failure.^10^, ^34^ Overall, the current body of evidence warrants the careful assessment of including failed SpO_2_ measurements as a referral indication.

Health-care policy, strategy, and clinical care guidelines are preferably informed by locally generated, contemporary, high-quality evidence. With respect to outpatient pulse oximetry, this translates to accounting for relevant patient factors as well as local context that might influence measurements.^28^, ^35^ This is a strength of our study. Projahnmo physicians were embedded within routine care systems at IMCI clinics and were trained and frequently supervised (by EDM) to follow a standardised SpO_2_ measurement approach we developed to facilitate objective, reproducible quality measurements and perfusion status criteria.^36^ Every 6 months we also evaluated pulse oximeter functionality using a reference artificial simulator and identified no issues. Lastly, we applied an altitude-adjusted hypoxaemic threshold previously derived from the fifth percentile of SpO_2_ values using the same device brand on 415 healthy children aged 3–11 months from the surveillance area, and therefore at similar altitude.^15^ This method resulted in a data-driven hypoxaemic threshold of less than 94%, which we applied in this work.^20^

There are five noteworthy limitations. First, our cohort was nested within a pneumococcal conjugate vaccine impact study of radiographic pneumonia that did not include infants younger than 3 months. Our findings might, therefore, underestimate hypoxaemia burden and mortality risk; however, they are still probably cautious, since an SpO_2_ of less than 94% was nevertheless common and mortality risk was markedly elevated. Both estimates would be expected to increase with the inclusion of children younger than 3 months. Although data are few, in Malawi, for example, 83 (35%) of 253 infants younger than 2 months seeking care at outpatients clinics had an SpO_2_ of less than 93%.^23^ Second, we conducted the study at IMCI clinics of upazila health complexes intended to care for patients who were mainly referred by health-care professionals. However, during the study period, we found 99·1% (3812 of 3848) of children attending the clinic were not referred. Our sensitivity analyses excluding all referred patients also did not alter our findings (appendix p 30). Third, because this study was conducted in an area under long-standing surveillance, it is possible that the presence of surveillance activities might lead to better health among children living in the surveilled area and attending IMCI clinics. Surveillance may also reduce case severity because of earlier care seeking. Thus, when extrapolating our findings to areas not under surveillance, it can be reasonably assumed that even higher mortality might occur, further accentuating the mortality and SpO_2_ associations we observed. Fourth, during this study period, approximately 10% of the surveillance population participated in a separate study evaluating weekly community health worker respiratory surveillance that included screening children for signs of acute respiratory illness and recommending care at IMCI clinics when screening positive. Although this activity appears to have modestly increased the volume of suspected pneumonia clinic cases (an additional 508 [13·2%] of 3848 participants were also under weekly surveillance), our post-hoc sensitivity analyses show this separate study did not meaningfully change any of our main findings because weekly surveillance cases had a similar distribution of both hypoxaemia strata and mortality compared with cases not participating in weekly surveillance (appendix p 41). Lastly, the fact that our follow-up approach leveraged an ongoing routine surveillance system limited our ability to collect antibiotic treatment adherence data. Although we would expect antibiotic adherence to be representative of a typical outpatient population in Bangladesh of approximately 75% treatment compliance (ie, missing <3 doses over a 5 day treatment course), and would not alter our findings, our results should be interpreted with this limitation in mind.^37^

In summary, despite notable differences in the child pneumonia context, epidemiology, and mortality between South Asia and sub-Saharan Africa, our findings on the implications of a higher SpO_2_ threshold and failed measurements in Bangladesh are consistent with previous work from Malawi. The WHO IMCI guidelines have had an important effect on child pneumonia mortality reductions over the past two decades.^38^, ^39^ However, WHO IMCI does not recommend hospital referral for a child in respiratory distress without a WHO IMCI danger sign and without pulse oximetry access,^9^ and our findings show that most fatalities might therefore be missed. We have two primary recommendations. First, that the SARS-CoV-2 pandemic has catalysed an unprecedented influx of respiratory care investment, including pulse oximeters, in LMICs such as Bangladesh. Our findings further stress the importance of these investments benefiting children, including outpatients. Further advocacy is needed. Second, we recommend re-evaluating the current SpO_2_ strategy recommended by the WHO IMCI outpatient pneumonia guidelines, which is also planned to be incorporated into the Bangladesh routine child health services.^12^ A higher SpO_2_ threshold or failed measurement, or both, should be considered for referral criteria, as should prioritising routine outpatient oximeter measurements on infants younger than 1 year since death is rare for children older than this. We observed high rates of hospitalisation refusal, which suggest that outpatient pulse oximeter interventions for hypoxaemic children that necessitate hospitalisation might have a lower than anticipated mortality effect in Bangladesh and similar LMIC settings. Further research is needed on hospital referral pathways and on children with an SpO_2_ of 90–93% or failed measurements, including clinical trials evaluating hospitalisation and oxygen treatment interventions on mortality.

## Data sharing

De-identified individual participant data and a data dictionary will be made available with publication, along with a signed data access agreement with the authors. Requests can be sent to emccoll3@jhmi.edu.

## Declaration of interests

We declare no competing interests.
