# Supplementary material for: Risk and accuracy of outpatient-identified hypoxaemia for death among suspected child pneumonia cases in rural Bangladesh: a multifacility prospective cohort study
Source: Lancet Respir Med. 2023 Sep;11(9):769–81. doi: 10.1016/S2213-2600(23)00098-X (PMC10469265; doi:10.1016/S2213-2600(23)00098-X)

THE LANCET
Respiratory Medicine

Supplementary appendix

This appendix formed part of the original submission and has been peer reviewed. We post it as supplied by the authors.

Supplement to: McCollum ED, Ahmed S, Roy AD, et al. Risk and accuracy of outpatient-identified hypoxaemia for death among suspected child pneumonia cases in rural Bangladesh: a multifacility prospective cohort study. *Lancet Respir Med* 2023; published online April 7. https://doi.org/10.1016/S2213-2600(23)00098-X.

Supplemental Material – Risk and accuracy of outpatient-identified hypoxemia for death among suspected child pneumonia cases in rural Bangladesh: a multi-facility cohort study

Corresponding author:
Dr. Eric D. McCollum, MD, MPH
Director, Johns Hopkins University Global Program in Pediatric Respiratory Sciences
Eudowood Division of Pediatric Respiratory Sciences, Johns Hopkins School of Medicine
Department of International Health, Johns Hopkins Bloomberg School of Public Health
Email: emccoll3@jhmi.edu

Contents

Section 1:
◊ **Panel: Key study definitions**

Section 2: Outpatient 3 – 11 month old children and three month mortality – secondary analysis
◊ **Supplemental Table 1: Three-month mortality of outpatient 3 – 11 month old children with suspected pneumonia in rural Bangladesh**
◊ **Supplemental Table 2: Risk of three-month mortality by SpO_2 measurement of outpatient 3 – 11 month old children with suspected pneumonia in rural Bangladesh**

Section 3: Outpatient 12 – 35 month old children: two-week and three-month mortality – secondary analysis
◊ **Supplemental Table 3: Characteristics of outpatient 12 – 35 month old children with suspected pneumonia in rural Bangladesh**
◊ **Supplemental Table 4: Two-week mortality of outpatient 12 – 35 month old children with suspected pneumonia in rural Bangladesh**
◊ **Supplemental Table 5: Risk of two-week mortality by SpO_2 measurement of outpatient 12 – 35 month old children with suspected pneumonia in rural Bangladesh**
◊ **Supplemental Table 6: WHO IMCI accuracy for identifying mortality with and without pulse oximetry and at varying SpO_2 thresholds for outpatient 12 – 35 month old children in Bangladesh**
◊ **Supplemental Figure 1: 3,240 children 12 – 35 months old with suspected pneumonia and SpO_2 measurement**
◊ **Supplemental Table 7: Three-month mortality of outpatient 12 – 35 month old children with suspected pneumonia in rural Bangladesh**
◊ **Supplemental Table 8: Risk of three-month mortality by SpO_2 measurement of outpatient 12 – 35 month old children with suspected pneumonia in rural Bangladesh**

Section 4: Outpatient 3-11 month old children and two-week mortality risk model specification – primary analysis
◊ **Model specification Summary Table (Supplemental Table 9) – Risk of two-week mortality by SpO_2 measurement of outpatient 3 – 11 month old children with suspected pneumonia in rural Bangladesh**
◊ **Model specification Statistical Output (Stata) – Risk of two-week mortality by SpO_2 measurement of outpatient 3 – 11 month old children with suspected pneumonia in rural Bangladesh**
◊ **Final model internal validation using nonparametric bootstrap estimation (Stata output)**

Section 5: Outpatient 3-11 month old children and three-month mortality risk model specification – secondary analysis
◊ **Model specification Summary Table (Supplemental Table 10) – Risk of three-month mortality by SpO_2 measurement of outpatient 3 – 11 month old children with suspected pneumonia in rural Bangladesh**
◊ **Model specification Statistical Output (Stata) – Risk of three-month mortality by SpO_2 measurement of outpatient 3 – 11 month old children with suspected pneumonia in rural Bangladesh**
(*note*: model specification for 12-35 month old children was not conducted due to sparse mortality observations at two-weeks (n=3) and three-months (n=5))

<u>Section 6:</u> Exploratory post-hoc sensitivity analyses

- ◊ **Supplemental Figure 2: Decision curve analysis**
- ◊ **Supplemental Table 11 – EMPIC eligibility criteria applied to outpatient 3 – 11 month olds in rural Bangladesh**
- ◊ **Supplemental Table 12 – Comparison of outpatient 3 – 11 month olds with difficulty breathing reported by caregivers and unreported by caregivers in rural Bangladesh**
- ◊ **Supplemental Table 13. Exclusion of all patients 3 – 11 month olds referred to outpatient clinics**
- ◊ **Exploratory post-hoc model specification – Risk of two-week mortality by SpO_2 measurement of outpatient 3 – 11 month old children with suspected pneumonia in rural Bangladesh**
    - o **Supplemental Table 14. Two-week mortality of outpatient 3 – 11 month old children with suspected pneumonia in rural Bangladesh – exploratory analysis of additional variables**
    - o **Supplemental Table 15. Summary Table**
    - o **Model specification of exploratory variables Statistical Output (Stata) – Risk of two-week mortality by SpO_2 measurement of outpatient 3 – 11 month old children with suspected pneumonia in rural Bangladesh**
- ◊ **Exploratory post-hoc sensitivity analysis – Weekly community surveillance for acute respiratory illness**
    - o **Supplemental Table 16. Hypoxemia strata**
    - o **Supplemental Table 17. Mortality**
    - o **Primary Model controlling for weekly surveillance (Stata output)**
    - o **Model selection accounting for weekly surveillance (Stata output)**
    - o **Subgroup analysis of Primary Model dropping 508 weekly surveillance cases (Stata output)**
- ◊ **Exploratory post-hoc sensitivity analysis – One month mortality**
    - o **Statistical output**
- ◊ **Exploratory post-hoc sensitivity analysis – Verbal autopsy data**
    - o **Supplemental Table 18. IMCI symptom VA results by probable LRI and SpO_2 strata among 3-11 month olds – 2 week mortality**
    - o **Statistical Output (Stata). Risk of two-week LRI mortality by IMCI symptom VA results among 3-11 month olds**
    - o **Supplemental Table 19. IMCI symptom VA results by probable LRI and SpO_2 strata among 3-11 month olds – 1 month mortality**
    - o **Statistical Output (Stata). Risk of 1 month LRI mortality by IMCI symptom VA results among 3-11 month olds**
    - o **Supplemental Table 20. IMCI symptom VA results by probable LRI and SpO_2 strata among 3-11 month olds – 3 month mortality**
    - o **Statistical Output (Stata). Risk of 3 month LRI mortality by IMCI symptom VA results among 3-11 month olds**
    - o **Supplemental Table 21. CCVA results by probable LRI and SpO_2 strata among 3-11 month olds – 2 week mortality**
    - o **Statistical Output (Stata). Risk of two-week LRI mortality by CCVA results among 3-11 month olds**
    - o **Supplemental Table 22. CCVA results by probable LRI and SpO_2 strata among 3-11 month olds – 1 month mortality**
    - o **Statistical Output (Stata). Risk of 1 month LRI mortality by CCVA results among 3-11 month olds**
    - o **Supplemental Table 23. CCVA results by probable LRI and SpO_2 strata among 3-11 month olds – 3 month mortality**
    - o **Statistical Output (Stata). Risk of 3 month LRI mortality by CCVA results among 3-11 month olds**
- ◊ **Exploratory post-hoc sensitivity analysis – Pneumococcal conjugate vaccine (PCV) data**
    - o **Supplemental Table 24. PCV status and death by two weeks**
    - o **Primary model controlling for participant PCV status (statistical output)**
    - o **Subgroup analysis of participants with a *known* PCV status (drops 1,014 participants and 13/24 mortality events) (statistical output)**
- ◊ **Exploratory post-hoc sensitivity analysis – <u>aRR by SpO_2 unit (1%) using the final model at two week mortality</u>**

- **Statistical output**
- **Supplemental Figure 3. Probability of mortality by SpO_2 1% units between 90-100%**

Section 1: Key study definitions

Panel. Key study definitions

**Eligibility criteria**	
**Inclusion criteria**	• 3-35 months of age *and*<br>• Residing in the surveillance area *and*<br>• Observed difficult breathing *and*<br>• Written informed consent
**Observed difficult breathing**	• fast breathing for age *or*<br>• chest indrawing *or*<br>• head nodding or tracheal tugging *or*<br>• persistent nasal flaring *or*<br>• cyanosis *or*<br>• stridor *or*<br>• grunting *or*<br>• adventitial lung auscultation sounds
**Fast breathing**	• respiratory rate 50 breaths/minute for 3–11 month olds<br>• respiratory rate 40 breaths/minute for 12–35 month olds
**WHO IMCI general danger sign**	• stridor at rest *or*<br>• observed or history of convulsions within prior 24 hours *or*<br>• not feeding or drinking *or*<br>• vomiting everything *or*<br>• lethargy or coma
**Severe acute malnutrition**	• Weight-for-age z score < -3 *or*<br>• bilateral pedal edema *or*<br>• Mid-upper arm circumference <11.5cm (children >6 months old)
**Quality SpO_2 measurement**	• Stable SpO_2 value for ≥3 seconds *and*<br>• ≥2 green bars on the device Quality Index scale *and*<br>• ≥2 green bars on the device Perfusion Index scale *and*<br>• <5 minutes measurement time
**WHO IMCI severe pneumonia**	• Observed difficult breathing *and*<br>• ≥1 WHO-defined general danger sign *or*<br>• Severe acute malnutrition
**WHO IMCI non-severe pneumonia**	• Observed difficult breathing *and*<br>• Fast breathing *or*<br>• Chest indrawing *and*<br>• Absence of any WHO-defined general danger signs *and*<br>• Absence of severe acute malnutrition

WHO indicates World Health Organization; SpO_2, peripheral oxyhemoglobin saturation; IMCI, Integrated Management of Childhood Illness

Section 2: Outpatient 3 – 11 month old children and three month mortality – secondary analysis

Supplemental Table 1. Three-month mortality of outpatient 3 – 11 month old children with suspected pneumonia in rural Bangladesh

**Characteristic**		**Alive, N=3,801**	**Dead, N=47**	**p value**
**Age, in months**	Median (IQR)	6 (4, 9)	5 (4, 7)	0.016
**Sex, n (%)**	Females	1,569 (41.2)	26 (55.3)	0.052
	Males	2,232 (58.7)	21 (44.6)	
**Clinic, n (%)**	Beanibazar	1,132 (29.7)	18 (38.3)	0.108
	Zakiganj	1,036 (27.2)	16 (34.0)	
	Kanaighat	1,633 (42.9)	13 (27.6)	
**Severe acute malnutrition[1], n (%)**		393 (7.7)	14 (29.7)	<0.001
**General danger signs[2], n (%)**		8 (0.2)	0 (0)	0.753
**SpO_2 in room air, n (%)**	94% – 100%	3,344 (87.9)	29 (61.7)	<0.001
	90% – 93%	297 (7.8)	9 (19.5)	
	< 90%, n (%)	98 (2.5)	4 (8.5)	
	Failed measurement, n	62 (1.6)	5 (10.6)	
**Hospitalization, n (%)**		344 (9.0)	14 (29.7)	<0.001
**Oxygen, n (%)**		147 (3.8)	10 (21.2)	<0.001

IQR indicates interquartile range; SpO2, peripheral arterial oxyhemoglobin saturation.
[1]Weight-for-age z score < -3 and/or a mid-upper arm circumference <11.5cm (for children >6 months old)
[2]Stridor at rest, convulsions, not feeding or drinking, vomiting everything, lethargy or coma

Supplemental Table 2. Risk of three-month mortality by SpO_2 measurement of outpatient 3 – 11-month-old children with suspected pneumonia in rural Bangladesh

Characteristic		Risk ratio[1]	95% CI	p value	Adjusted risk ratio[1]	95% CI	p value
**WHO IMCI and no SpO_2, N=3,848**	WHO IMCI referral criteria[2]	4.7	2.5, 8.8	<0.001	–	–	–
**WHO IMCI and SpO_2 (continuous), N=3,781**	WHO IMCI referral criteria[2]	4.7	2.5, 8.8	<0.001	4.1	2.1, 8.2	<0.001
	SpO_2	0.9	0.8, 0.9	<0.001	0.9	0.9, 0.9	<0.001
**WHO IMCI and SpO_2 (strata), N=3,848**	WHO IMCI referral criteria[2]	4.7	2.5, 8.8	<0.001	4.1	2.2, 7.7	<0.001
	90% – 93%	3.4	1.6, 7.1	0.001	3.1	1.5, 6.4	0.002
	< 90%	4.5	1.6, 12.7	0.004	4.1	1.4, 11.9	0.009
	Failed measurement	8.6	3.4, 21.7	<0.001	7.5	3.0, 18.6	<0.001
**SpO_2 <90% or failed measurement threshold, N=3,848**	WHO IMCI referral criteria[2]	4.7	2.5, 8.8	<0.001	4.4	2.2, 9.4	<0.001
	< 90% or failed measurement	5.1	2.5, 10.4	<0.001	4.5	2.2, 9.5	<0.001
**SpO_2 <94% or failed measurement threshold, N=3,848**	WHO IMCI referral criteria[2]	4.7	2.5, 8.8	<0.001	4.2	2.2, 7.8	<0.001
	< 94% or failed measurement	4.4	2.4, 7.8	<0.001	3.9	2.2, 7.1	<0.001

CI indicates confidence interval; SpO_2, peripheral oxyhemoglobin saturation; CI, confidence interval.

[1]Risk ratio estimated by poisson regression with robust variance estimation

[2]WHO IMCI referral criteria, which includes any of: stridor at rest, convulsions, not feeding or drinking, vomiting everything, lethargy or coma, or severe acute malnutrition (weight-for-age z score < -3 and/or a mid-upper arm circumference <11.5cm (for children >6 months old))

Section 3: Outpatient 12 – 35 month old children: two-week and three-month mortality – secondary analysis

Supplemental Table 3. Characteristics of outpatient 12 – 35 month old children with suspected pneumonia in rural Bangladesh

Characteristic		No pneumonia, N=392	Non-severe pneumonia, N=2,406	Severe pneumonia, N=442	Total, N=3,240
**Age, in months**	**Median (IQR)**	20 (15,26)	20 (15,26)	22 (16,27)	20 (15,26)
**Sex, n (%)**	**Females**	175 (44.6)	1,060 (44.0)	183 (41.4)	1,418 (43.70
**Clinic, n (%)**	**Beanibazar**	240 (61.2)	775 (32.20	86 (19.4)	1,101 (33.9)
	**Zakiganj**	76 (19.3)	573 (23.8)	141 (31.9)	790 (24.3)
	**Kanaighat**	76 (19.3)	1,058 (43.9)	215 (48.6)	1,349 (41.6)
**Weight, in kg**	**Mean (SD)**	9.7 (1.5)	9.5 (1.4)	7.4 (1.0)	9.2 (1.6)
**Weight-for-age z score**	**$\geq$-2.0**	302 (77.0)	1,677 (69.7)	9 (2.0)	1,988 (61.3)
	**<-2.0 to $\geq$-3.0**	90 (22.9)	729 (30.3)	9 (2.0)	828 (25.5)
	**<-3.0**	–	–	424 (95.9)	424 (13.0)
**Mid-upper arm circumference <11.5cm, n (%)**		–	–	16 (3.6)	16 (0.4)
**Severe acute malnutrition, n (%)**		–	–	429 (97.0)	429 (13.2)
**Fever $\geq$101 °F, n (%)**		61 (15.5)	668 (27.7)	117 (26.4)	846 (26.1)
**Fast breathing for age, n (%)**		–	2,325 (96.6)	395 (89.3)	2,720 (83.9)
**Lower chest wall indrawing, n (%)**		–	711 (29.5)	140 (31.6)	851 (26.2)
**Respiratory distress, n (%)**		4 (1.0)	193 (8.0)	43 (9.7)	240 (7.4)
	**Head nodding, n (%)**	–	101 (4.2)	27 (6.1)	128 (3.9)
	**Nasal flaring, n (%)**	4 (1.0)	115 (4.7)	26 (5.8)	145 (4.4)
	**Wheeze, n (%)**	5 (1.2)	37 (1.5)	10 (2.2)	52 (1.6)
	**Grunting, n (%)**	1 (0.2)	6 (0.2)	3 (0.6)	10 (0.3)
**WHO IMCI danger signs, n (%)**		–	–	14 (3.1)	14 (0.4)
	**Stridor, n (%)**	–	–	–	–
	**Convulsion, n (%)**	–	–	13 (2.9)	13 (0.4)
	**Not feeding, n (%)**	–	–	–	–
	**Vomiting everything, n (%)**	–	–	–	–
	**Lethargy, n (%)**	–	–	2 (0.4)	2 (0)
**SpO_2 in room air[1]**	**Median (IQR)**	97 (96, 99) (n=387)	97 (96, 99) (n=2,358)	97 (96, 98) (n=425)	97 (96, 99) n=3,170
	**94% – 100%, n (%)**	363 (92.6)	2,198 (91.3)	390 (88.2)	2,951 (91.0)
	**90% – 93%, n (%)**	22 (5.6)	133 (5.5)	27 (6.1)	182 (5.6)
	**< 90%, n (%)**	2 (0.5)	27 (1.1)	8 (1.8)	37 (1.1)
	**Failed measurement, n (%)**	5 (1.2)	48 (2.0)	17 (3.8)	70 (2.1)
**Hospitalization, n (%)**		6 (1.5)	116 (4.8)	37 (8.3)	159 (4.9)
**Oxygen, n (%)**		3 (0.7)	43 (1.7)	15 (3.3)	61 (1.8)
**Mortality, n (%)**	**Two weeks**	–	2 (0.1)	1 (0.2)	3 (0.1)
	**Three months**	–	3 (0.1)	2 (0.4)	5 (0.1)

WHO indicates World Health Organization; IMCI, Integrated Management of Childhood Illness; IQR, interquartile range; SD, standard deviation; SpO2, peripheral arterial oxyhemoglobin saturation.
[1]387 measurements for no pneumonia category; 2,358 measurements for non-severe pneumonia category; 425 measurements for severe pneumonia category; 3,170 measurements total.

Supplemental Table 4. Two-week mortality of outpatient 12 – 35 month old children with suspected pneumonia in rural Bangladesh

**Characteristic**		**Alive, N=3,237**	**Dead, N=3**	**p value**
**Age, in months**	**Median (IQR)**	20 (15, 26)	13 (12, 17)	0.049
**Sex, n (%)**	**Females**	1,417 (43.7)	1 (33.3)	0.716
	**Males**	1,820 (56.2)	2 (66.6)	
**Clinic, n (%)**	**Beanibazar**	1,101 (34.0)	0 (–)	0.010
	**Zakiganj**	787 (24.3)	3 (100.0)	
	**Kanaighat**	1,349 (41.6)	0 (–)	
**Severe acute malnutrition[1], n (%)**		428 (13.2)	1 (33.3)	0.304
**WHO IMCI danger signs[2], n (%)**		14 (0.4)	0 (–)	0.909
**SpO_2 in room air, n (%)**	**94% – 100%**	2,950 (91.1)	1 (33.3)	<0.001
	**90% – 93%**	181 (5.5)	1 (33.3)	
	**< 90%, n (%)**	37 (1.1)	0 (–)	
	**Failed measurement, n**	69 (2.1)	1 (33.3)	
**Hospitalization, n (%)**		157 (4.8)	2 (66.6)	<0.001
**Oxygen, n (%)**		59 (1.8)	2 (66.6)	<0.001

IQR indicates interquartile range; SpO2, peripheral arterial oxyhemoglobin saturation.
[1]Weight-for-age z score < -3 and/or a MUAC <11.5cm (for children >6 months old)
[2]Stridor at rest, convulsions, not feeding or drinking, vomiting everything, lethargy or coma

Supplemental Table 5. Risk of two-week mortality by SpO_2 measurement of outpatient 12 – 35 month old children with suspected pneumonia in rural Bangladesh

Characteristic		Risk ratio[1]	95% CI	p value
WHO IMCI and no SpO_2, N=3,240	WHO IMCI referral criteria[2]	3.1	0.2, 34.8	0.346
SpO_2 (continuous), N=3,170	SpO_2	0.9	0.8, 1.0	0.165
WHO IMCI and SpO_2 (strata), N=3,240	90% – 93%	16.2	1.0, 258.2	0.049
	< 90%	–	–	–
	Failed measurement	42.1	2.6, 667.4	0.008
SpO_2 <90% or failed measurement threshold, N=3,240	< 90% or failed measurement	14.6	1.3, 160.2	0.028
SpO_2 <94% or failed measurement threshold, N=3,240	< 94% or failed measurement	20.4	1.8, 224.6	0.014

CI indicates confidence interval; SpO_2, peripheral oxyhemoglobin saturation; CI, confidence interval.

[1]Risk ratio estimated by poisson regression with robust variance estimation

[2]WHO IMCI referral criteria, which includes any of: stridor at rest, convulsions, not feeding or drinking, vomiting everything, lethargy or coma, or severe acute malnutrition (weight-for-age z score < -3 and/or a mid-upper arm circumference <11.5cm (for children >6 months old))

Supplemental Table 6. WHO IMCI accuracy for identifying two-week mortality with and without pulse oximetry and at varying SpO_2 thresholds for outpatient 12 – 35 month old children in Bangladesh

Model	Referral eligibility prevalence	Sensitivity (95% CI)	Specificity (95% CI)	PPV (95% CI)	NPV (95% CI)	LR Positive (95% CI)	LR Negative (95% CI)	AUC	Diagnostic OR (95% CI)	LRT[1]
**WHO IMCI without pulse oximetry**	13.6%	33.3% (0.8%, 90.6%)	86.4% (85.1%, 87.5%)	0.2% (0%, 1.2%)	99.9% (99.7%, 99.9%)	2.4 (0.4, 12.2)	0.7 (0.3, 1.7)	0.59 (0.2, 0.9)	3.1 (0.2, 35.0)	Ref
**WHO IMCI with SpO_2 <90%**	14.5%	33.3% (0.8%, 90.6%)	85.5% (84.2%, 86.7%)	0.2% (0%, 1.1%)	99.9% (99.7%, 100%)	2.3 (0.4, 11.4)	0.7 (0.3, 1.7)	0.59 (0.2, 0.9)	2.9 (0.2, 32.5)	0.1
**WHO IMCI with SpO_2 <90% or failure**	16.1%	33.3% (0.8%, 90.6%)	83.8% (82.5%, 85.1%)	0.1% (0%, 1.0%)	99.9% (99.7%, 100%)	2.0 (0.4, 10.2)	0.7 (0.3, 1.7)	0.58 (0.2, 0.9)	2.5 (0.2, 28.6)	2.8
**WHO IMCI with SpO_2 <94%**	19.3%	66.7% (9.4%, 99.2%)	81.6% (80.2%, 82.9%)	0.3% (0%, 1.2%)	100% (99.8%, 100%)	3.6 (1.6, 8.1)	0.4 (0, 2.0)	0.74 (0.4, 1.0)	2.8 (1.2, 6.6)	0.1
**WHO IMCI with SpO_2 <94% or failure**	20.9%	66.6% (9.4%, 99.1%)	79.0% (77.6%, 80.4%)	2.0% (1.1%, 3.3%)	99.7% (99.4%, 99.8%)	3.1 (1.4, 7.1)	0.4 (0, 2.0)	0.72 (0.4, 1.0)	7.5 (0.6, 83.5)	5.8

WHO indicates World Health Organization; IMCI, Integrated Management of Childhood Illnesses; SpO_2, peripheral oxyhemoglobin saturation; CI, confidence interval; PPV, positive predictive value; NPV, negative predictive value; OR, odds ratio.

[1]Chi-square test statistic for the likelihood-ratio test comparing the reduced model (IMCI without pulse oximetry) and the full model (IMCI with pulse oximetry (SpO_2 threshold specified by table row)). P values for LRT comparisons as follows: IMCI with SpO_2 <90%= 0.7797; SpO_2 <90% or failure=0.0892; IMCI with SpO_2 <94%=0.1814; IMCI with SpO_2 <94% or failure=0.0160.

Supplemental Figure 1: 3,240 children 12 – 35 months old with suspected pneumonia and SpO_2 measurement

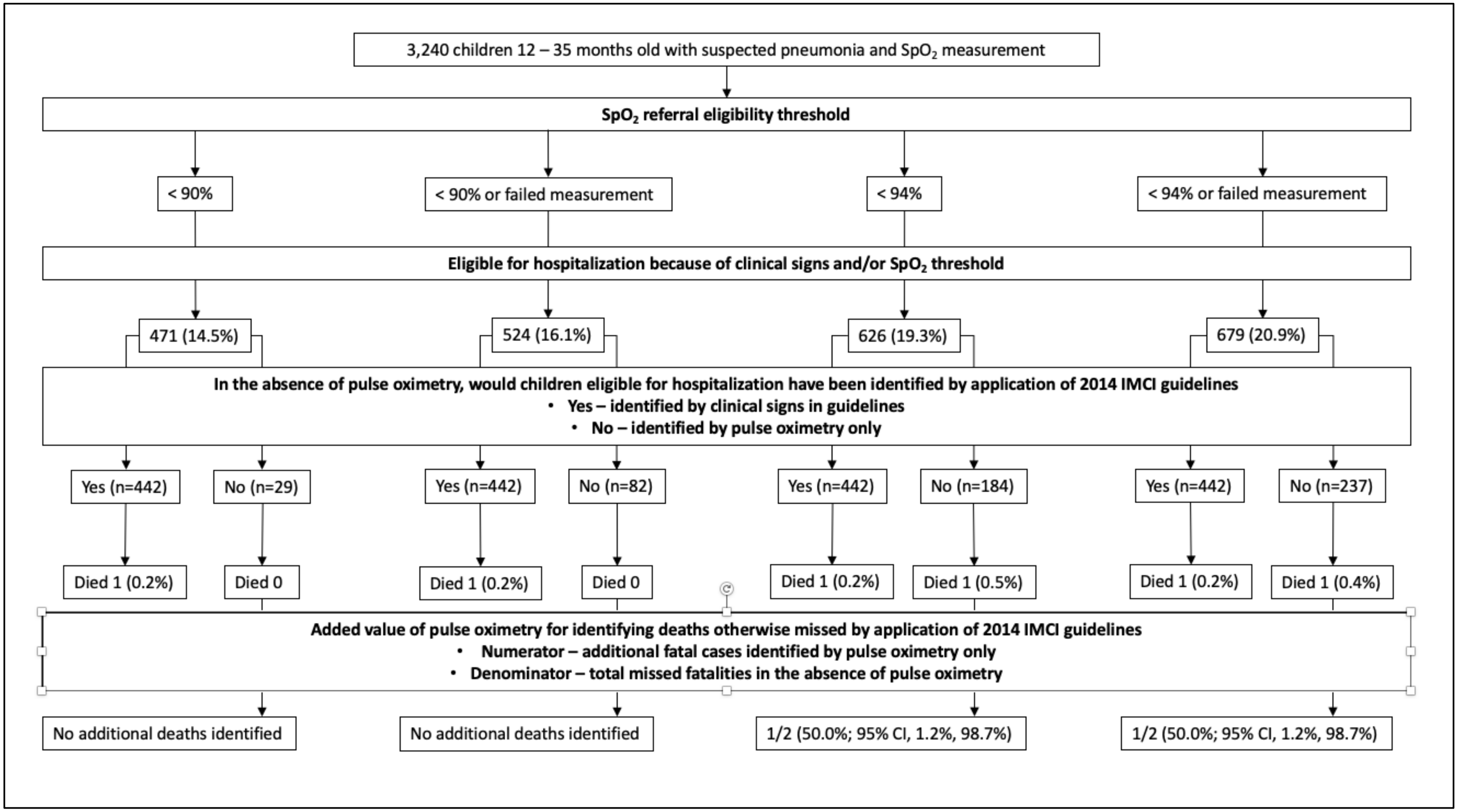

Supplemental Table 7. Three-month mortality of outpatient 12 – 35 month old children with suspected pneumonia in rural Bangladesh

Characteristic		Alive, N=3,235	Dead, N=5	p value
**Age, in months**	**Median (IQR)**	20 (15, 26)	16 (13, 17)	0.134
**Sex, n (%)**	**Females**	1,416 (43.7)	2 (40.0)	0.865
	**Males**	1,819 (56.2)	3 (60.0)	
**Clinic, n (%)**	**Beanibazar**	1,100 (34.0)	1 (20.0)	0.178
	**Zakiganj**	787 (24.3)	3 (60.0)	
	**Kanaighat**	1,348 (41.6)	1 (20.0)	
**Severe acute malnutrition[1], n (%)**		427 (13.2)	2 (40.0)	0.077
**WHO IMCI danger signs[2], n (%)**		14 (0.4)	0 (–)	0.883
**SpO_2 in room air, n (%)**	**94% – 100%**	2,949 (91.1)	2 (40.0)	<0.001
	**90% – 93%**	181 (5.6)	1 (20.0)	
	**< 90%, n (%)**	36 (1.1)	1 (20.0)	
	**Failed measurement, n**	69 (2.1)	1 (20.0)	
**Hospitalization, n (%)**		156 (4.8)	2 (60.0)	<0.001
**Oxygen, n (%)**		59 (1.8)	2 (40.0)	<0.001

IQR indicates interquartile range; SpO2, peripheral arterial oxyhemoglobin saturation.
[1]Weight-for-age z score < -3 and/or a MUAC <11.5cm (for children >6 months old)
[2]Stridor at rest, convulsions, not feeding or drinking, vomiting everything, lethargy or coma

Supplemental Table 8: Risk of three-month mortality by SpO_2 measurement of outpatient 12 – 35 month old children with suspected pneumonia in rural Bangladesh

Characteristic		Risk ratio[1]	95% CI	p value
**WHO IMCI and no SpO_2, N=3,240**	**WHO IMCI referral criteria[2]**	4.2	0.7, 25.1	0.114
**WHO IMCI and SpO_2 (continuous) , N=3,170**	**SpO_2**			
**WHO IMCI and SpO_2 (strata), N=3,240**	**90% – 93%**	8.1	0.7, 89.0	0.087
	**< 90%**	39.8	3.6, 430.3	0.002
	**Failed measurement**	21.0	1.9, 229.8	0.012
**SpO_2 <90% or failed measurement threshold, N=3,240**	**< 90% or failed measurement**	19.5	3.2, 115.6	0.001
**SpO_2 <94% or failed measurement threshold, N=3,240**	**< 94% or failed measurement**	15.3	2.5, 91.3	0.003

CI indicates confidence interval; SpO_2, peripheral oxyhemoglobin saturation; CI, confidence interval.

[1]Risk ratio estimated by poisson regression with robust variance estimation

[2]WHO IMCI referral criteria, which includes any of: stridor at rest, convulsions, not feeding or drinking, vomiting everything, lethargy or coma, or severe acute malnutrition (weight-for-age z score < -3 and/or a mid-upper arm circumference <11.5cm (for children >6 months old))

Section 4: Outpatient 3-11 month old children and two-week mortality risk model specification – primary analysis

Overall approach: Due to the fact that mortality events at two-weeks were modest in number (n=24) for our primary analysis of 3 – 11 month olds (and also for secondary analyses) the model specification approach sought to optimize both model fit and parsimony. Our research question of interest was whether a low oxygen saturation was independently associated with an elevated mortality risk when controlling for the currently recommended Integrated Management of Childhood Illnesses (IMCI) criteria (i.e., World Health Organization (WHO) IMCI danger signs and/or severe acute malnutrition). That is, does oxygen saturation have added value in predicting fatalities when employed in an outpatient context in Bangladesh?

We therefore employed a forward selection approach where variables with a p value <0.05 in bivariable analysis (see Table 2) of the association between mortality and exposure variables were added stepwise to the base model of hypoxemia and IMCI referral criteria. We then compared the Bayesian Information Criterion (BIC) score of the 'nested model' (i.e., saved model) and the 'non-nested model' (i.e., current model). As described by Williams in 2022 (see https://www3.nd.edu/~rwilliam/xsoc73994/L05.pdf) and Jorgensen et al (see https://methods.sagepub.com/foundations/goodness-of-fit-measures) BIC evaluates model fit while also comparing the nested and non-nested models. Per Williams and Jorgensen BIC favors the model that is most likely to have produced the observed data, while also having penalties for models with variables that do not lead to an improved fit. Overall, this results in BIC favoring more parsimonious but suitable models.

To facilitate BIC model comparison we utilized a scale proposed by Raftery (https://www3.nd.edu/~rwilliam/xsoc73994/L05.pdf) that categorizes the absolute magnitude of BIC difference with a qualitative description of the strength of difference. We used Raftery's scale to facilitate decision-making on model selection.

Raftery scale:

Absolute difference	Evidence
0-2	Weak
2-6	Positive
6-10	Strong
>10	Very strong

Whenever evidence of the absolute difference in BIC values between models was 'weak' we also considered the p value of the added term. In this scenario of 'weak' BIC evidence (i.e., neither model favored) we then evaluated the added term's p value. If the added term's p value was <0.05 then we added the variable to the model. If the added term's p value was >0.05 then we did not add the variable to the model and retained the nested (saved) model. A similar approach was utilized for secondary analyses of mortality risk as mortality events remained modest in number.

Model specification Summary Table – Risk of two-week mortality by SpO_2 measurement of outpatient 3 – 11-month-old children with suspected pneumonia in rural Bangladesh – primary analysis

Model A: exposure variables	Model B: exposure variables	BIC – Model A	BIC – Model B	BIC – absolute difference	Evidence per Raftery scale	Favored Model
• WHO IMCI referral criteria<br>• SpO_2 <90%<br>• SpO_2 90-93%<br>• Failed SpO_2	Model A plus:<br>• clinic (as fixed effect)	-31444.231	-31424.047	20.1	Very strong	Model A
	Model A plus: hospital (as interaction term)	-31391.621	-31444.231	52.6	Very strong	Model A
	Model A plus: oxygen (as interaction term)	-31393.801	-31444.231	50.4	Very strong	Model A
	Model A plus: Severe acute malnutrition (as interaction term)	-31385.667	-31444.231	58.5	Very strong	Model A
	Model A plus: WHO IMCI referral criteria (as interaction term)	-31385.593	-31444.231	58.6	Very strong	Model A

Model specification Statistical Output (Stata) – Risk of two-week mortality by SpO_2 measurement of outpatient 3 – 11 month old children with suspected pneumonia in rural Bangladesh

- **Clinic as fixed effect:**

Model A:

```
Poisson regression                              Number of obs     =      3,848
                                                Wald chi2(4)      =      45.28
                                                Prob > chi2       =     0.0000
Log pseudolikelihood = -132.20513               Pseudo R2         =     0.0936

------------------------------------------------------------------------------
             |               Robust
     death2w |        IRR   Std. Err.      z    P>|z|     [95% Conf. Interval]
-------------+----------------------------------------------------------------
  IMCI_refer |
         yes |   3.088078   1.488237     2.34   0.019     1.200794    7.941595
             |
     hypoxia |
       90-93 |   4.297255   2.224147     2.82   0.005      1.55823    11.85088
         <90 |   10.25691   6.015159     3.97   0.000     3.249602    32.37451
      failed |   11.39268   7.505195     3.69   0.000       3.1324    41.43571
             |
       _cons |   .0030693   .0009125   -19.46   0.000     .0017139    .0054966
------------------------------------------------------------------------------
Note: _cons estimates baseline incidence rate.
```

Model B:

```
Poisson regression                              Number of obs     =      3,848
                                                Wald chi2(6)      =      52.99
                                                Prob > chi2       =     0.0000
Log pseudolikelihood = -129.91435               Pseudo R2         =     0.1093

------------------------------------------------------------------------------
             |               Robust
     death2w |        IRR   Std. Err.      z    P>|z|     [95% Conf. Interval]
-------------+----------------------------------------------------------------
  IMCI_refer |
         yes |   3.104556   1.522976     2.31   0.021     1.186951     8.12019
             |
     hypoxia |
       90-93 |   3.525215   1.950332     2.28   0.023     1.191947    10.42592
         <90 |   8.823905   5.215732     3.68   0.000     2.770277    28.10596
      failed |   9.585936   6.372919     3.40   0.001     2.604587    35.28012
             |
      clinic |
    Zakiganj |   .8068364   .3608843    -0.48   0.631      .335782    1.938713
   Kanaighat |   .3160922    .191415    -1.90   0.057     .0964615    1.035794
             |
       _cons |   .0048353   .0019283   -13.37   0.000     .0022129    .0105652
------------------------------------------------------------------------------
Note: _cons estimates baseline incidence rate.

Measures of Fit for poisson of death2w
```

	Model B	Model A	Difference
Model:	poisson	poisson	
N:	3848	3848	0
BIC:	-31424.047	-31444.231	20.184

Difference of 20.184 in BIC provides very strong support for Model A.

- **Clinic as random effect:**

```
Mixed-effects Poisson regression                Number of obs     =      3,848
Group variable: clinic                          Number of groups  =          3

                                                Obs per group:
                                                              min =      1,052
                                                              avg =    1,282.7
                                                              max =      1,646

Integration points =   7                        Wald chi2(4)      =      32.03
Log likelihood = -132.11966                     Prob > chi2       =     0.0000

------------------------------------------------------------------------------
     death2w |        IRR   Std. Err.      z    P>|z|     [95% Conf. Interval]
-------------+----------------------------------------------------------------
     hypoxia |
      90-93  |   4.064415   2.234161     2.55   0.011     1.383893    11.93696
        <90  |   9.839038   5.779364     3.89   0.000     3.111461    31.11293
     failed  |   10.85599   7.145203     3.62   0.000     2.988305    39.43791
             |
  IMCI_refer |   3.089452   1.466933     2.38   0.018     1.218187    7.835178
       _cons |   .0030839   .0010222   -17.44   0.000     .0016105    .0059053
------------------------------------------------------------------------------
Note: _cons estimates baseline incidence rate (conditional on zero random effects).

------------------------------------------------------------------------------
  Random-effects Parameters  |   Estimate   Std. Err.     [95% Conf. Interval]
-----------------------------+------------------------------------------------
clinic: Identity             |
                   sd(_cons) |   .2256012   .3461003      .0111556    4.562368
------------------------------------------------------------------------------
LR test vs. Poisson model: chibar2(01) = 0.17         Prob >= chibar2 = 0.3396
```

As Prob >= chibar2 = 0.3396 the model with clinic as random effect is not favored. <u>Model A</u> retained.

- **Hospital as interaction term:**

<u>Model A:</u>

```
Poisson regression                              Number of obs     =      3,848
                                                Wald chi2(4)      =      45.28
                                                Prob > chi2       =     0.0000
Log pseudolikelihood = -132.20513               Pseudo R2         =     0.0936

------------------------------------------------------------------------------
             |               Robust
     death2w |        IRR   Std. Err.      z    P>|z|     [95% Conf. Interval]
-------------+----------------------------------------------------------------
  IMCI_refer |
        yes  |   3.088078   1.488237     2.34   0.019     1.200794    7.941595
             |
     hypoxia |
      90-93  |   4.297255   2.224147     2.82   0.005      1.55823    11.85088
        <90  |   10.25691   6.015159     3.97   0.000     3.249602    32.37451
     failed  |   11.39268   7.505195     3.69   0.000       3.1324    41.43571
             |
       _cons |   .0030693   .0009125   -19.46   0.000     .0017139    .0054966
------------------------------------------------------------------------------
Note: _cons estimates baseline incidence rate.
```

<u>Model B:</u>

```
Poisson regression                              Number of obs     =      3,848
                                                Wald chi2(7)      =          .
                                                Prob > chi2       =          .
Log pseudolikelihood = -129.61626               Pseudo R2         =     0.1113

--------------------------------------------------------------------------------------
                     |               Robust
             death2w |        IRR   Std. Err.      z    P>|z|     [95% Conf. Interval]
---------------------+----------------------------------------------------------------
             hypoxia |
```

90-93	3.956257	2.557308	2.13	0.033	1.114478	14.04422
<90	10.71148	8.328808	3.05	0.002	2.333405	49.17098
failed	6.97e-07	2.24e-07	-44.07	0.000	3.71e-07	1.31e-06
IMCI_refer	2.733921	1.41045	1.95	0.051	.9945916	7.514967
hypoxia#hosp_admit						
94-100#Hospitalized	2.712538	2.174594	1.24	0.213	.5636149	13.05477
90-93#Hospitalized	1.853218	1.711559	0.67	0.504	.3032427	11.32564
<90#Hospitalized	1.177236	1.167856	0.16	0.869	.1684396	8.227779
failed#Hospitalized	3.30e+07	1.94e+07	29.43	0.000	1.04e+07	1.04e+08
_cons	.0028118	.0008777	-18.82	0.000	.0015251	.0051842

Note: _cons estimates baseline incidence rate.

Measures of Fit for poisson of death2w

	Model B	Model A	Difference
Model:	poisson	poisson	
N:	3848	3848	0
BIC:	-31391.621	-31444.231	52.609

Difference of 52.609 in BIC provides very strong support for Model A.

- **Oxygen as interaction term:**

Model A:

```
Poisson regression                              Number of obs     =      3,848
                                                Wald chi2(4)      =      45.28
                                                Prob > chi2       =     0.0000
Log pseudolikelihood = -132.20513               Pseudo R2         =     0.0936
```

death2w	IRR	Robust Std. Err.	z	P>|z|	[95% Conf.	Interval]
IMCI_refer						
yes	3.088078	1.488237	2.34	0.019	1.200794	7.941595
hypoxia						
90-93	4.297255	2.224147	2.82	0.005	1.55823	11.85088
<90	10.25691	6.015159	3.97	0.000	3.249602	32.37451
failed	11.39268	7.505195	3.69	0.000	3.1324	41.43571
_cons	.0030693	.0009125	-19.46	0.000	.0017139	.0054966

Note: _cons estimates baseline incidence rate.

Model B:

```
Poisson regression                              Number of obs     =      3,848
                                                Wald chi2(8)      =      61.53
                                                Prob > chi2       =     0.0000
Log pseudolikelihood = -128.52644               Pseudo R2         =     0.1188
```

death2w	IRR	Robust Std. Err.	z	P>|z|	[95% Conf.	Interval]
hypoxia						
90-93	4.672748	2.723364	2.65	0.008	1.490996	14.64429
<90	7.614756	5.973938	2.59	0.010	1.636328	35.43575
failed	6.911972	7.263007	1.84	0.066	.8813958	54.20421
IMCI_refer	2.835139	1.404959	2.10	0.035	1.073395	7.488399
hypoxia#oxygen_treat1						
94-100#oxygen treatment	7.487596	5.954273	2.53	0.011	1.575619	35.58226
90-93#oxygen treatment	1.613031	1.731597	0.45	0.656	.1967314	13.22549
<90#oxygen treatment	3.831333	3.786484	1.36	0.174	.5522205	26.58198
failed#oxygen treatment	3.692888	4.504832	1.07	0.284	.3380753	40.33842

_cons	.0026705	.0008379	-18.89	0.000	.0014438	.0049393

Note: _cons estimates baseline incidence rate.

Measures of Fit for poisson of death2w

	**Model B**	**Model A**	**Difference**
Model:	poisson	poisson	
N:	3848	3848	0
BIC:	-31393.801	-31444.231	50.430

Difference of 50.430 in BIC provides very strong support for Model A.

- **Severe acute malnutrition as interaction term**

Model A:

Poisson regression

Log pseudolikelihood = -132.20513

Number of obs	= 3,848
Wald chi2(4)	= 45.28
Prob > chi2	= 0.0000
Pseudo R2	= 0.0936

death2w	IRR	Robust Std. Err.	z	P>|z|	[95% Conf.	Interval]
IMCI_refer						
yes	3.088078	1.488237	2.34	0.019	1.200794	7.941595
hypoxia						
90-93	4.297255	2.224147	2.82	0.005	1.55823	11.85088
<90	10.25691	6.015159	3.97	0.000	3.249602	32.37451
failed	11.39268	7.505195	3.69	0.000	3.1324	41.43571
_cons	.0030693	.0009125	-19.46	0.000	.0017139	.0054966

Note: _cons estimates baseline incidence rate.

Model B:

Poisson regression

Log pseudolikelihood = -128.46604

Number of obs	= 3,848
Wald chi2(7)	= 2987.25
Prob > chi2	= 0.0000
Pseudo R2	= 0.1192

death2w	IRR	Robust Std. Err.	z	P>|z|	[95% Conf.	Interval]
hypoxia						
90-93	2.562591	1.996971	1.21	0.227	.5563606	11.80326
<90	15.26281	9.028405	4.61	0.000	4.787664	48.65701
failed	17.95996	11.7362	4.42	0.000	4.989708	64.64508
wazrefer						
SAM	4.200377	2.786909	2.16	0.031	1.144259	15.41886
hypoxia#wazrefer						
90-93#SAM	2.765048	3.080908	0.91	0.361	.3113623	24.55497
<90#SAM	5.93e-09	5.20e-09	-21.58	0.000	1.06e-09	3.31e-08
failed#SAM	5.04e-09	4.69e-09	-20.51	0.000	8.12e-10	3.13e-08
_cons	.0028799	.0009587	-17.57	0.000	.0014997	.0055303

Note: _cons estimates baseline incidence rate.

Measures of Fit for poisson of death2w

	**Model B**	**Model A**	**Difference**
Model:	poisson	poisson	
N:	3848	3848	0
BIC:	-31385.667	-31444.231	58.564

Difference of 58.564 in BIC provides very strong support for Model A

- **WHO IMCI referral as interaction term**

Model A:

```
Poisson regression                              Number of obs     =      3,848
                                                Wald chi2(4)      =      45.28
                                                Prob > chi2       =     0.0000
Log pseudolikelihood = -132.20513               Pseudo R2         =     0.0936

------------------------------------------------------------------------------
             |               Robust
     death2w |        IRR   Std. Err.      z    P>|z|     [95% Conf. Interval]
-------------+----------------------------------------------------------------
  IMCI_refer |
        yes  |   3.088078   1.488237     2.34   0.019     1.200794    7.941595
             |
     hypoxia |
      90-93  |   4.297255   2.224147     2.82   0.005      1.55823    11.85088
        <90  |   10.25691   6.015159     3.97   0.000     3.249602    32.37451
     failed  |   11.39268   7.505195     3.69   0.000       3.1324    41.43571
             |
       _cons |   .0030693   .0009125   -19.46   0.000     .0017139    .0054966
------------------------------------------------------------------------------
Note: _cons estimates baseline incidence rate.
```

Model B:

```
Poisson regression                              Number of obs     =      3,848
                                                Wald chi2(7)      =    3066.45
                                                Prob > chi2       =     0.0000
Log pseudolikelihood = -128.50282               Pseudo R2         =     0.1190

--------------------------------------------------------------------------------------
                     |               Robust
             death2w |        IRR   Std. Err.      z    P>|z|     [95% Conf. Interval]
--------------------+-----------------------------------------------------------------
             hypoxia |
              90-93  |   2.555211   1.991219     1.20   0.229     .5547591    11.76926
                <90  |   15.38797   9.100837     4.62   0.000     4.827911    49.04599
             failed  |   17.90824   11.70239     4.42   0.000     4.975345    64.45885
                     |
          IMCI_refer |
                yes  |   4.041524   2.681982     2.10   0.035     1.100734    14.83912
                     |
  hypoxia#IMCI_refer |
          90-93#yes  |   2.873729   3.202202     0.95   0.343     .3235565    25.52357
            <90#yes  |   6.09e-09   5.32e-09   -21.66   0.000     1.10e-09    3.38e-08
         failed#yes  |   5.24e-09   4.88e-09   -20.47   0.000     8.44e-10    3.25e-08
                     |
               _cons |   .0028882   .0009615   -17.56   0.000      .001504    .0055463
--------------------------------------------------------------------------------------
Note: _cons estimates baseline incidence rate.

Measures of Fit for poisson of death2w

                             Model B           Model A        Difference
Model:                       poisson           poisson
N:                              3848              3848                 0
BIC:                      -31385.593        -31444.231            58.638
```

Difference of 58.638 in BIC provides very strong support for Model A.

◊ **Final model internal validation using nonparametric bootstrap estimation** (Stata output)

```
bootstrap, reps (100): poisson death2w i.IMCI_refer i.hypoxia, vce(robust) irr
(running poisson on estimation sample)

Bootstrap replications (100)
----+--- 1 ---+--- 2 ---+--- 3 ---+--- 4 ---+--- 5
..................................................    50
..................................................   100

Poisson regression                              Number of obs     =      3,848
                                                Replications      =        100
                                                Wald chi2(2)      =      28.22
                                                Prob > chi2       =     0.0000
Log pseudolikelihood = -133.45167               Pseudo R2         =     0.0850

------------------------------------------------------------------------------
             |   Observed   Bootstrap                         Normal-based
     death2w |        IRR   Std. Err.      z    P>|z|     [95% Conf. Interval]
-------------+----------------------------------------------------------------
  IMCI_refer |
        yes  |   3.128901   1.532008     2.33   0.020     1.198441    8.168963
             |
     hypoxia |
<94 or fail  |   6.597115   2.941184     4.23   0.000     2.753365    15.80681
       _cons |   .0030611   .0010239   -17.31   0.000     .0015892    .0058965
------------------------------------------------------------------------------
Note: _cons estimates baseline incidence rate.
```

Section 5: Outpatient 3-11 month old children and three-month mortality risk model specification – secondary analysis

Model specification Summary Table – Risk of *three-month* mortality by SpO_2 measurement of outpatient 3 – 11 month old children with suspected pneumonia in rural Bangladesh

Model A: exposure variables	Model B: exposure variables	BIC – Model A	BIC – Model B	BIC – absolute difference	Evidence per Raftery scale	Favored Model
• **WHO IMCI referral criteria**<br>• **SpO_2 <90%**<br>• **SpO_2 90-93%**<br>• **Failed SpO_2**	**Model A plus:**<br>• **clinic (as fixed effect)**	-31138.405	-31115.271	23.1	Very strong	Model A
	**Model A plus: hospital (as interaction term)**	-31138.405	-31075.272	63.1	Very strong	Model A
	**Model A plus: oxygen (as interaction term)**	-31138.405	-31078.613	59.7	Very strong	Model A
	**Model A plus: Severe acute malnutrition (as interaction term)**	-31138.405	-31075.541	62.8	Very strong	Model A
	**Model A plus: WHO IMCI referral criteria (as interaction term)**	-31138.405	-31075.541	62.8	Very strong	Model A

Model specification Statistical Output (Stata) – Risk of *three-month* mortality by SpO_2 measurement of outpatient 3 – 11 month old children with suspected pneumonia in rural Bangladesh

- **Clinic as fixed effect:**

Model A:

Poisson regression	Number of obs	=	3,837
	Wald chi2(3)	=	30.80
	Prob > chi2	=	0.0000
Log pseudolikelihood = -242.48427	Pseudo R2	=	0.0450

death3m	IRR	Robust Std. Err.	z	P>|z|	[95% Conf.	Interval]
hypoxia						
90-93	3.421933	1.289732	3.26	0.001	1.634754	7.162932
<90	4.547667	2.382444	2.89	0.004	1.628767	12.69751
failed	8.654143	4.052849	4.61	0.000	3.456171	21.6697
_cons	.0086233	.0015946	-25.71	0.000	.0060016	.01239

Note: _cons estimates baseline incidence rate.

Measures of Fit for poisson of death3m

Model B:

Poisson regression	Number of obs	=	3,837
	Wald chi2(6)	=	65.12
	Prob > chi2	=	0.0000
Log pseudolikelihood = -233.42017	Pseudo R2	=	0.0807

death3m	IRR	Robust Std. Err.	z	P>|z|	[95% Conf.	Interval]
hypoxia						
90-93	2.79589	1.072758	2.68	0.007	1.318019	5.930872
<90	3.781961	2.039606	2.47	0.014	1.314212	10.8835
failed	6.833077	3.206039	4.10	0.000	2.724192	17.13937
IMCI_refer						
yes	4.218072	1.335886	4.54	0.000	2.267431	7.846826
clinic						
Zakiganj	.887668	.3024481	-0.35	0.727	.4552264	1.730907
Kanaighat	.5795922	.2091498	-1.51	0.131	.2857331	1.175667
_cons	.0089308	.0024943	-16.89	0.000	.0051661	.0154391

Note: _cons estimates baseline incidence rate.

Measures of Fit for poisson of death3m

	**Model B**	**Model A.**	**Difference**
Model:	poisson	poisson	
N:	3837	3837	0
BIC:	-31115.271	-31138.405	23.134

Difference of 23.134 in BIC provides very strong support for Model A.

- **Clinic as random effect:**

Mixed-effects Poisson regression	Number of obs	=	3,837
Group variable: clinic	Number of groups	=	3
	Obs per group:		
	min	=	1,049
	avg	=	1,279.0
	max	=	1,643

Integration points = 7 — Wald chi2(4) = 51.00
Log likelihood = -234.6181 — Prob > chi2 = 0.0000

death3m	IRR	Std. Err.	z	P>|z|	[95% Conf.	Interval]
hypoxia						
90-93	3.116741	1.192995	2.97	0.003	1.471927	6.599562
<90	4.114381	2.198526	2.65	0.008	1.443655	11.72588
failed	7.538975	3.665533	4.15	0.000	2.907025	19.55131
IMCI_refer	4.17394	1.340478	4.45	0.000	2.224229	7.832727
_cons	.00694	.00141	-24.46	0.000	.0046603	.0103347

Note: _cons estimates baseline incidence rate (conditional on zero random effects).

Random-effects Parameters	Estimate	Std. Err.	[95% Conf.	Interval]
clinic: Identity				
sd(_cons)	2.03e-09	.3071851	0	.

LR test vs. Poisson model: chibar2(01) = 0.00 — Prob >= chibar2 = 1.0000

As Prob >= chibar2 = 1.0000 the model with clinic as random effect is not favored. Model A retained.

- **Hospital as interaction term:**

Model A:

Poisson regression — Number of obs = 3,837
Wald chi2(3) = 30.80
Prob > chi2 = 0.0000
Log pseudolikelihood = -242.48427 — Pseudo R2 = 0.0450

death3m	IRR	Robust Std. Err.	z	P>|z|	[95% Conf.	Interval]
hypoxia						
90-93	3.421933	1.289732	3.26	0.001	1.634754	7.162932
<90	4.547667	2.382444	2.89	0.004	1.628767	12.69751
failed	8.654143	4.052849	4.61	0.000	3.456171	21.6697
_cons	.0086233	.0015946	-25.71	0.000	.0060016	.01239

Note: _cons estimates baseline incidence rate.

Measures of Fit for poisson of death3m

Model B:

Poisson regression — Number of obs = 3,837
Wald chi2(8) = 71.50
Prob > chi2 = 0.0000
Log pseudolikelihood = -232.78855 — Pseudo R2 = 0.0832

death3m	IRR	Robust Std. Err.	z	P>|z|	[95% Conf.	Interval]
hypoxia						
90-93	2.610541	1.253465	2.00	0.046	1.018642	6.690205
<90	4.159782	3.07086	1.93	0.053	.9787941	17.67868
failed	3.842992	3.617086	1.43	0.153	.6074422	24.31274
IMCI_refer						
yes	3.797708	1.260521	4.02	0.000	1.981499	7.278623
hypoxia#hosp_admit						
94-100#Hospitalized	2.036664	1.113455	1.30	0.193	.6975304	5.946695
90-93#Hospitalized	2.053398	1.35302	1.09	0.275	.5644186	7.470416

<90#Hospitalized	1.159271	1.158979	0.15	0.882	.1633795	8.225686
failed#Hospitalized	2.985105	3.139161	1.04	0.298	.3800398	23.44716
_cons	.0066056	.0013647	-24.30	0.000	.0044061	.0099029

Note: _cons estimates baseline incidence rate.

Measures of Fit for poisson of death3m

	Model B	Model A	Difference
Model:	poisson	poisson	
N:	3837	3837	0
BIC:	-31075.272	-31138.405	63.133

Difference of 63.133 in BIC provides very strong support for Model A.

- **Oxygen as interaction term:**

Model A:

Poisson regression

	Number of obs	=	3,837
	Wald chi2(3)	=	30.80
	Prob > chi2	=	0.0000
Log pseudolikelihood = -242.48427	Pseudo R2	=	0.0450

death3m	IRR	Robust Std. Err.	z	P>|z|	[95% Conf.	Interval]
hypoxia						
90-93	3.421933	1.289732	3.26	0.001	1.634754	7.162932
<90	4.547667	2.382444	2.89	0.004	1.628767	12.69751
failed	8.654143	4.052849	4.61	0.000	3.456171	21.6697
_cons	.0086233	.0015946	-25.71	0.000	.0060016	.01239

Note: _cons estimates baseline incidence rate.

Measures of Fit for poisson of death3m

Model B:

Poisson regression

	Number of obs	=	3,837
	Wald chi2(8)	=	79.38
	Prob > chi2	=	0.0000
Log pseudolikelihood = -231.11796	Pseudo R2	=	0.0898

death3m	IRR	Robust Std. Err.	z	P>|z|	[95% Conf.	Interval]
hypoxia						
90-93	3.087967	1.285479	2.71	0.007	1.365617	6.982589
<90	2.818841	2.090287	1.40	0.162	.6589755	12.0579
failed	5.16432	3.608703	2.35	0.019	1.312847	20.31479
IMCI_refer						
yes	3.909904	1.287935	4.14	0.000	2.050112	7.456836
hypoxia#oxygen_treat1						
94-100#oxygen treatment	4.085228	2.560228	2.25	0.025	1.196093	13.953
90-93#oxygen treatment	1.708854	1.333272	0.69	0.492	.3703268	7.885417
<90#oxygen treatment	3.921632	3.915643	1.37	0.171	.5540707	27.75674
failed#oxygen treatment	2.65533	2.293471	1.13	0.258	.4885576	14.43183
_cons	.0065034	.0013177	-24.85	0.000	.0043718	.0096742

Note: _cons estimates baseline incidence rate.

Measures of Fit for poisson of death3m

	Model B	Model A	Difference
Model:	poisson	poisson	

N:	3837	3837	0
BIC:	-31078.613	-31138.405	59.792

Difference of 59.792 in BIC provides very strong support for Model A.

- ## Severe acute malnutrition as interaction term

Model A:

Poisson regression

	Number of obs	=	3,837
	Wald chi2(3)	=	30.80
	Prob > chi2	=	0.0000
Log pseudolikelihood = -242.48427	Pseudo R2	=	0.0450

death3m	IRR	Robust Std. Err.	z	P>|z|	[95% Conf.	Interval]
hypoxia						
90-93	3.421933	1.289732	3.26	0.001	1.634754	7.162932
<90	4.547667	2.382444	2.89	0.004	1.628767	12.69751
failed	8.654143	4.052849	4.61	0.000	3.456171	21.6697
_cons	.0086233	.0015946	-25.71	0.000	.0060016	.01239

Note: _cons estimates baseline incidence rate.

Measures of Fit for poisson of death3m

Model B:

Poisson regression

	Number of obs	=	3,837
	Wald chi2(7)	=	3784.10
	Prob > chi2	=	0.0000
Log pseudolikelihood = -232.65386	Pseudo R2	=	0.0837

death3m	IRR	Robust Std. Err.	z	P>|z|	[95% Conf.	Interval]
hypoxia						
90-93	2.738977	1.352003	2.04	0.041	1.04093	7.207012
<90	6.573545	3.517065	3.52	0.000	2.303449	18.75948
failed	7.650213	4.612302	3.37	0.001	2.346851	24.938
IMCI_refer						
yes	4.604039	1.88962	3.72	0.000	2.059602	10.29188
hypoxia#IMCI_refer						
90-93#yes	1.340438	1.026362	0.38	0.702	.2988744	6.011801
<90#yes	2.88e-10	2.01e-10	-31.36	0.000	7.29e-11	1.14e-09
failed#yes	.9331607	.8722026	-0.07	0.941	.1494029	5.828459
_cons	.0067611	.0014706	-22.97	0.000	.0044144	.0103552

Note: _cons estimates baseline incidence rate.

Measures of Fit for poisson of death3m

	Model B	Model A	Difference
Model:	poisson	poisson	
N:	3837	3837	0
BIC:	-31075.541	-31138.405	62.864

Difference of 13.349 in BIC' provides very strong support for saved model.

Difference of 62.864 in BIC provides very strong support for Model A.

- **WHO IMCI referral as interaction term**

Model A:

Poisson regression	Number of obs	=	3,837
	Wald chi2(3)	=	30.80
	Prob > chi2	=	0.0000
Log pseudolikelihood = -242.48427	Pseudo R2	=	0.0450

death3m	IRR	Robust Std. Err.	z	P>|z|	[95% Conf.	Interval]
hypoxia						
90-93	3.421933	1.289732	3.26	0.001	1.634754	7.162932
<90	4.547667	2.382444	2.89	0.004	1.628767	12.69751
failed	8.654143	4.052849	4.61	0.000	3.456171	21.6697
_cons	.0086233	.0015946	-25.71	0.000	.0060016	.01239

Note: _cons estimates baseline incidence rate.

Measures of Fit for poisson of death3m

Model B:

Poisson regression	Number of obs	=	3,837
	Wald chi2(7)	=	3784.10
	Prob > chi2	=	0.0000
Log pseudolikelihood = -232.65386	Pseudo R2	=	0.0837

death3m	IRR	Robust Std. Err.	z	P>|z|	[95% Conf.	Interval]
hypoxia						
90-93	2.738977	1.352003	2.04	0.041	1.04093	7.207012
<90	6.573545	3.517065	3.52	0.000	2.303449	18.75948
failed	7.650213	4.612302	3.37	0.001	2.346851	24.938
IMCI_refer						
yes	4.604039	1.88962	3.72	0.000	2.059602	10.29188
hypoxia#IMCI_refer						
90-93#yes	1.340438	1.026362	0.38	0.702	.2988744	6.011801
<90#yes	2.88e-10	2.01e-10	-31.36	0.000	7.29e-11	1.14e-09
failed#yes	.9331607	.8722026	-0.07	0.941	.1494029	5.828459
_cons	.0067611	.0014706	-22.97	0.000	.0044144	.0103552

Note: _cons estimates baseline incidence rate.

Measures of Fit for poisson of death3m

	Model B	Model A	Difference
Model:	poisson	poisson	
N:	3837	3837	0
BIC:	-31075.541	-31138.405	62.864

Difference of 62.864 in BIC provides very strong support for Model A.

<u>Section 6: </u>Exploratory post-hoc sensitivity analyses

◊ **Supplemental Figure 2:** Decision curve analysis

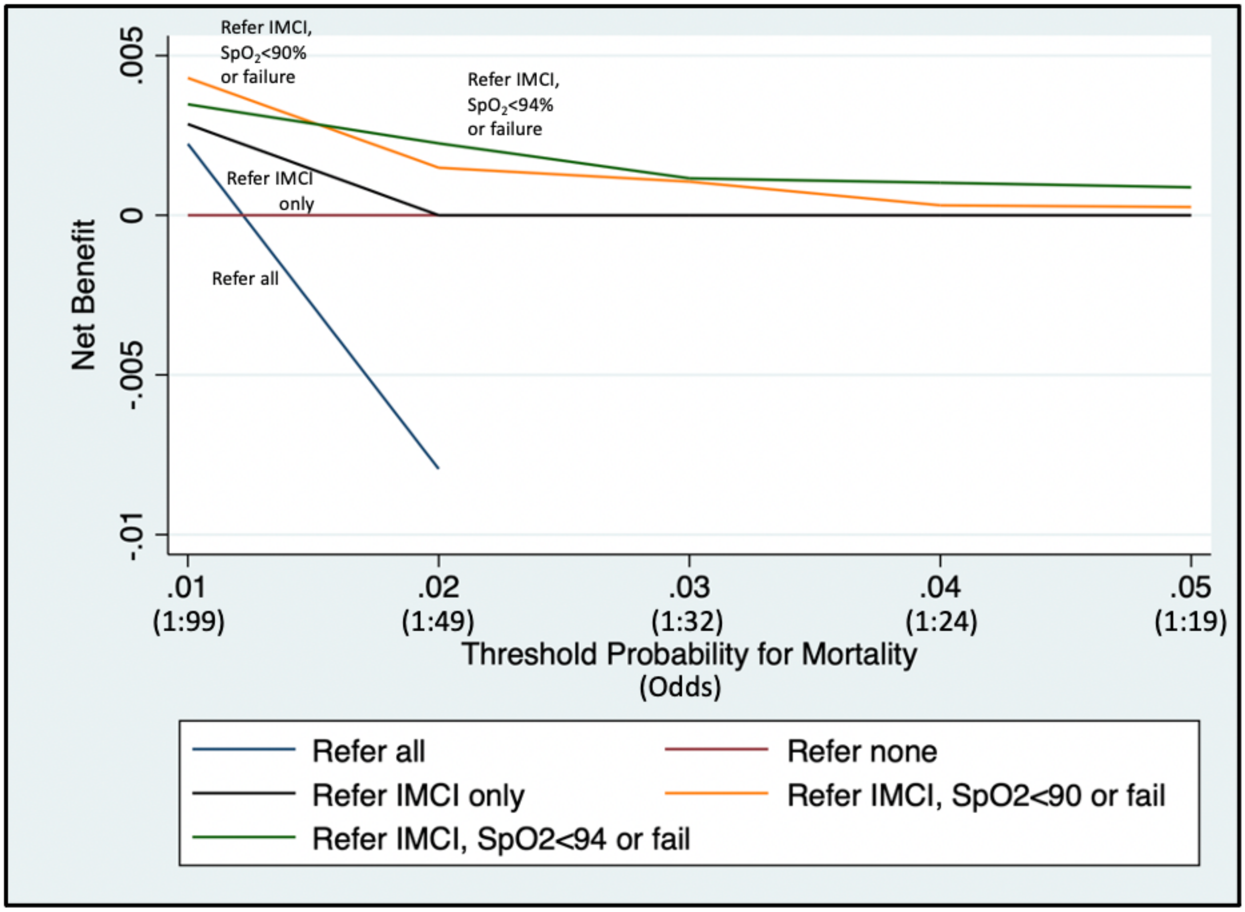

At all threshold probabilities for mortality an approach incorporating pulse oximetry (**yellow or green lines**) has an approximately 2-3x higher net benefit (identifying deaths amongst all referrals) than use of IMCI alone (**black line**). Above a threshold probability of 1.5%, an approach using a SpO_2 <94% threshold or failure (**green line**) has a roughly 2x higher net benefit than an approach using a <90% threshold or failure for referral (**yellow line**). In other words, an approach using IMCI, SpO_2 <94%, or failed measurement for referral is the favored referral approach when missing a death event would be valued as 65.6 times worse (or lower) than not referring a patient. **This decision curve analysis overall favors the primary model.**

◊ **Supplemental Table 11. Exploratory post-hoc analysis: EMPIC eligibility criteria applied to outpatient 3 – 11 month olds in rural Bangladesh**

a) Mortality at two weeks

	EMPIC criteria	Total
alive	1,044<br>99.52	1,044<br>99.52
dead	5<br>0.48	5<br>0.48
Total	1,049<br>100.00	1,049<br>100.00

◊ **Supplemental Table 12. Exploratory post-hoc analysis: Comparison of outpatient 3 – 11 month olds with difficult breathing reported by caregivers and unreported by caregivers in rural Bangladesh**

	**Mortality at two weeks**		
**Difficult breathing**	alive	dead	**Total**
Caregiver reported	3,609<br>99.48	19<br>**0.52**	3,628<br>100.00
Not reported by caregiver but observed by study physician	215<br>97.73	5<br>**2.27**	220<br>100.00
Total	3,824<br>99.38	24<br>0.62	3,848<br>100.00

Pearson chi2(1) = 10.2373 **Pr = 0.001**

◊ **Supplemental Table 13. Exploratory post-hoc analysis: Exclusion of all patients 3 – 11 month olds referred to outpatient clinics**

```
       refer |      Freq.     Percent        Cum.
-------------+-----------------------------------
not referred |      3,812       99.06       99.06
    referred |         36        0.94      100.00
-------------+-----------------------------------
       Total |      3,848      100.00
```

**Characteristic N=3,812**		**Risk ratio**[1]	**95% CI**	**p value**	**Adjusted risk ratio**[2]	**95% CI**	**p value**
**SpO_2 strata**	**94% – 100%**	Ref			Ref		
	**90% – 93%**	4.0	1.2, 12.5	0.017	3.7	1.2, 11.1	0.020
	**< 90%**	12.3	3.9, 38.0	<0.001	11.4	3.5, 36.7	<0.001
	**Failed measurement**	17.2	4.9, 60.1	<0.001	14.9	3.9, 56.2	<0.001

CI indicates confidence interval; SpO_2, peripheral oxyhemoglobin saturation; CI, confidence interval.
[1]Risk ratio estimated by poisson models with robust variance estimation
[2]Model adjusted for WHO IMCI referral criteria, which includes any of: stridor at rest, convulsions, not feeding or drinking, vomiting everything, lethargy or coma, or severe acute malnutrition (weight-for-age z score < -3 and/or a mid-upper arm circumference <11.5cm (for children >6 months old))

◊ Exploratory post-hoc model specification – Risk of two-week mortality by SpO_2 measurement of outpatient 3 – 11 month old children with suspected pneumonia in rural Bangladesh

Additional variables examined in post-hoc analyses:

- Age in months (continuous)
- Sex (binary)
- Duration of illness (in days)
- Distance from clinic (access to care)
- PCV (pneumococcal conjugate vaccine) status
- Maternal age in years (continuous)
- Wealth quintile (socioeconomic status)
- Cooking source (indoor air pollution)
- Water source
- Type of toilet (sanitation)

The following variables were not available in our dataset:

- Prior hospitalization
- Chronic medical conditions
- Birth weight
- Gestational age
- Anemia (hemoglobin levels)

Supplemental Table 14. Two-week mortality of outpatient 3 – 11 month old children with suspected pneumonia in rural Bangladesh – exploratory analysis of additional variables

Characteristic		Alive, N=3,824	Dead, N=24	p value
**Illness duration, in days**[1]	Median (IQR)	3 (3,5) (n=3,755)	3 (3, 5) (n=23)	0.9918
**Distance from clinic, km**	Median (IQR)	8.9 (4.4, 13.9)	10.6 (7.0, 16.5)	0.2078
**PCV status, n (%)**	Zero doses	189 (4.9%)	1 (4.1%)	0.008
	≥1 dose	2,634 (68.8%)	10 (41.6%)	
	Missing	1,001 (26.1%)	13 (54.1%)	
**Maternal age, in years**	Mean (SD)	27.8 (6.2)	30.0 (7.4)	0.0845
**Wealth quintile, n (%)**[2]	First (Poorest)	678 (17.7%)	5 (20.8%)	0.941
	Second	687 (17.9%)	5 (20.8%)	
	Middle	714 (18.6%)	5 (20.8%)	
	Fourth	727 (19.0%)	5 (20.8%)	
	Fifth (Richest)	728 (19.0%)	3 (12.5%)	
**Cooking fuel, n (%)**[3]	Biomass	3,341 (87.3%)	22 (91.6%)	0.789
	No Biomass	186 (4.8%)	1 (4.1%)	
**Water source, n (%)**[4]	Piped water	37 (0.8%)	0	0.269
	Well water	2,272 (59.4%)	19 (79.1%)	
	Surface water	1,226 (32.0%)	4 (16.6%)	
**Type of toilet, n (%)**[5]	Flush	1,475 (38.5%)	9 (37.5%)	0.864
	Pit	2,022 (52.8%)	14 (58.3%)	
	None	36 (0.9%)	0	

IQR indicates interquartile range.
[1]70 participants with missing illness duration data (69 alive, 1 death)
[2]291 participants with missing wealth quintile data (290 alive, 1 death)
[3]298 participants with missing cooking fuel data (297 alive, 1 death)
[4]290 participants with missing cooking fuel data (289 alive, 1 death)
[5]292 participants with missing cooking fuel data (291 alive, 1 death)

Supplemental Table 15. Summary Table

Model A: exposure variables	Model B: exposure variables	BIC – Model A	BIC – Model B	BIC – absolute difference	Evidence per Raftery scale	Favored Model
• **WHO IMCI referral criteria** • **SpO_2 <90%** • **SpO_2 90-93%** • **Failed SpO_2**	**Model A plus:** • **agemonths**	-31444.231	-31438.108	6.1	Strong	Model A
• **WHO IMCI referral criteria** • **SpO_2 <90%** • **SpO_2 90-93%** • **Failed SpO_2**	**Model A plus:** • **sex**	-31444.231	-31436.671	7.5	Strong	Model A
• **WHO IMCI referral criteria** • **SpO_2 <90%** • **SpO_2 90-93%** • **Failed SpO_2**	**Model A plus:** • **Duration of illness**	-31444.231	-30797.799	646.4	Very Strong	Model A
• **WHO IMCI referral criteria** • **SpO_2 <90%** • **SpO_2 90-93%** • **Failed SpO_2**	**Model A plus: Distance from clinic**	-31444.231	-31437.309	6.9	Strong	Model A
• **WHO IMCI referral criteria** • **SpO_2 <90%** • **SpO_2 90-93%** • **Failed SpO_2**	**Model A plus: Mothers age in years**	-31444.231	-28513.890	2930.3	Very Strong	Model A
• **WHO IMCI referral criteria** • **SpO_2 <90%** • **SpO_2 90-93%** • **Failed SpO_2**	**Model A plus: Wealth quintile**	-31444.231	-28765.905	2678.3	Very Strong	Model A
• **WHO IMCI referral criteria** • **SpO_2 <90%** • **SpO_2 90-93%** • **Failed SpO_2**	**Model A plus: Cooking fuel**	-31444.231	-28701.620	2742.6	Very Strong	Model A
• **WHO IMCI referral criteria** • **SpO_2 <90%** • **SpO_2 90-93%** • **Failed SpO_2**	**Model A plus: Drinking water source**	-31444.231	-28776.592	2667.6	Very Strong	Model A
• **WHO IMCI referral criteria** • **SpO_2 <90%** • **SpO_2 90-93%** • **Failed SpO_2**	**Model A plus: Toilet**	-31444.231	-28756.691	2687.5	Very Strong	Model A

Model specification of exploratory variables Statistical Output (Stata) – Risk of two-week mortality by SpO_2 measurement of outpatient 3 – 11 month old children with suspected pneumonia in rural Bangladesh

- **Age in months (continuous)**

Model A:

```
Poisson regression                                Number of obs     =      3,848
                                                  Wald chi2(4)      =      45.28
                                                  Prob > chi2       =     0.0000
Log pseudolikelihood = -132.20513                 Pseudo R2         =     0.0936

------------------------------------------------------------------------------
             |               Robust
     death2w |        IRR   Std. Err.      z    P>|z|     [95% Conf. Interval]
-------------+----------------------------------------------------------------
  IMCI_refer |
        yes  |   3.088078   1.488237     2.34   0.019     1.200794    7.941595
             |
     hypoxia |
      90-93  |   4.297255   2.224147     2.82   0.005      1.55823    11.85088
        <90  |   10.25691   6.015159     3.97   0.000     3.249602    32.37451
     failed  |   11.39268   7.505195     3.69   0.000       3.1324    41.43571
             |
       _cons |   .0030693   .0009125   -19.46   0.000     .0017139    .0054966
------------------------------------------------------------------------------
Note: _cons estimates baseline incidence rate.
```

Model B:

```
Poisson regression                                Number of obs     =      3,848
                                                  Wald chi2(5)      =      45.97
                                                  Prob > chi2       =     0.0000
Log pseudolikelihood = -131.13879                 Pseudo R2         =     0.1009

------------------------------------------------------------------------------
             |               Robust
     death2w |        IRR   Std. Err.      z    P>|z|     [95% Conf. Interval]
-------------+----------------------------------------------------------------
  IMCI_refer |
        yes  |   3.140531   1.519488     2.37   0.018     1.216648    8.106646
             |
     hypoxia |
      90-93  |   4.044673   2.087875     2.71   0.007     1.470585     11.1244
        <90  |   9.544046   5.410533     3.98   0.000     3.141848    28.99211
     failed  |   10.92275   7.398166     3.53   0.000     2.896001    41.19698
             |
   agemonths |   .8826595   .0812026    -1.36   0.175     .7370291    1.057065
       _cons |   .0066092   .0038579    -8.60   0.000     .0021052    .0207496
------------------------------------------------------------------------------
Note: _cons estimates baseline incidence rate.

                                Model B           Model A         Difference
Model:                          poisson           poisson
N:                                 3848              3848                  0
BIC:                         -31438.108        -31444.231              6.123
```

Difference of 6.123 in BIC provides strong support for Model A.

- **Sex**

Model A:

```
Poisson regression                                Number of obs     =      3,848
                                                  Wald chi2(4)      =      45.28
                                                  Prob > chi2       =     0.0000
Log pseudolikelihood = -132.20513                 Pseudo R2         =     0.0936

------------------------------------------------------------------------------
             |               Robust
     death2w |        IRR   Std. Err.      z    P>|z|     [95% Conf. Interval]
-------------+----------------------------------------------------------------
```

```
 IMCI_refer |
        yes |   3.088078   1.488237     2.34   0.019     1.200794    7.941595
            |
    hypoxia |
      90-93 |   4.297255   2.224147     2.82   0.005      1.55823    11.85088
        <90 |   10.25691   6.015159     3.97   0.000     3.249602    32.37451
     failed |   11.39268   7.505195     3.69   0.000       3.1324    41.43571
            |
      _cons |   .0030693   .0009125   -19.46   0.000     .0017139    .0054966
------------------------------------------------------------------------------
Note: _cons estimates baseline incidence rate.
```

Model B:

```
Poisson regression                              Number of obs     =      3,848
                                                Wald chi2(5)      =      50.38
                                                Prob > chi2       =     0.0000
Log pseudolikelihood = -131.85725               Pseudo R2         =     0.0960

------------------------------------------------------------------------------
             |               Robust
     death2w |        IRR   Std. Err.      z    P>|z|     [95% Conf. Interval]
-------------+----------------------------------------------------------------
  IMCI_refer |
         yes |   3.195236   1.547166     2.40   0.016     1.236922     8.25398
             |
     hypoxia |
       90-93 |   4.178833   2.247738     2.66   0.008     1.456147    11.99236
         <90 |   10.13321   5.947302     3.95   0.000     3.207498    32.01308
      failed |   11.20877    7.44957     3.64   0.000     3.046723    41.23664
             |
         sex |   1.409442    .588582     0.82   0.411     .6217077    3.195276
       _cons |   .0026267   .0008145   -19.16   0.000     .0014304    .0048235
------------------------------------------------------------------------------
Note: _cons estimates baseline incidence rate.

Measures of Fit for poisson of death2w
```

	**Model B**	**Model B**	**Difference**
Model:	poisson	poisson	
N:	3848	3848	0
BIC:	-31436.671	-31444.231	7.560

Difference of 7.560 in BIC provides strong support for <u>Model A</u>.

- **Duration of illness (in days)**

Model A:

```
Poisson regression                              Number of obs     =      3,848
                                                Wald chi2(4)      =      45.28
                                                Prob > chi2       =     0.0000
Log pseudolikelihood = -132.20513               Pseudo R2         =     0.0936

------------------------------------------------------------------------------
             |               Robust
     death2w |        IRR   Std. Err.      z    P>|z|     [95% Conf. Interval]
-------------+----------------------------------------------------------------
  IMCI_refer |
         yes |   3.088078   1.488237     2.34   0.019     1.200794    7.941595
             |
     hypoxia |
       90-93 |   4.297255   2.224147     2.82   0.005      1.55823    11.85088
         <90 |   10.25691   6.015159     3.97   0.000     3.249602    32.37451
      failed |   11.39268   7.505195     3.69   0.000       3.1324    41.43571
             |
       _cons |   .0030693   .0009125   -19.46   0.000     .0017139    .0054966
------------------------------------------------------------------------------
Note: _cons estimates baseline incidence rate.
```

Model B:

```
Poisson regression                              Number of obs     =      3,778
                                                Wald chi2(5)      =      42.10
                                                Prob > chi2       =     0.0000
```

```
Log pseudolikelihood =  -127.7515              Pseudo R2         =     0.0897

------------------------------------------------------------------------------
             |               Robust
     death2w |        IRR   Std. Err.      z    P>|z|     [95% Conf. Interval]
-------------+----------------------------------------------------------------
  IMCI_refer |
        yes  |   3.261268   1.596386     2.41   0.016     1.249466    8.512328
             |
     hypoxia |
      90-93  |   4.441332   2.301493     2.88   0.004     1.608502    12.26323
        <90  |   7.756679   5.130963     3.10   0.002     2.121362      28.362
     failed  |   12.33339   8.227027     3.77   0.000     3.336462      45.591
             |
    symp_dur |    .953101    .077059    -0.59   0.552      .813427    1.116758
       _cons |   .0036975   .0014294   -14.49   0.000     .0017331    .0078882
------------------------------------------------------------------------------
Note: _cons estimates baseline incidence rate.

Measures of Fit for poisson of death2w
```

	Model B	Model A	Difference
Model:	poisson	poisson	
N:	3778	3848	-70
BIC:	-30797.799	-31444.231	646.432

Difference of 646.432 in BIC provides very strong support for Model A.

- **Distance from clinic (access to care)**

Model A:

```
Poisson regression                              Number of obs     =      3,848
                                                Wald chi2(4)      =      45.28
                                                Prob > chi2       =     0.0000
Log pseudolikelihood = -132.20513               Pseudo R2         =     0.0936

------------------------------------------------------------------------------
             |               Robust
     death2w |        IRR   Std. Err.      z    P>|z|     [95% Conf. Interval]
-------------+----------------------------------------------------------------
  IMCI_refer |
        yes  |   3.088078   1.488237     2.34   0.019     1.200794    7.941595
             |
     hypoxia |
      90-93  |   4.297255   2.224147     2.82   0.005      1.55823    11.85088
        <90  |   10.25691   6.015159     3.97   0.000     3.249602    32.37451
     failed  |   11.39268   7.505195     3.69   0.000       3.1324    41.43571
             |
       _cons |   .0030693   .0009125   -19.46   0.000     .0017139    .0054966
------------------------------------------------------------------------------
Note: _cons estimates baseline incidence rate.
```

Model B:

```
Poisson regression                              Number of obs     =      3,848
                                                Wald chi2(5)      =      46.02
                                                Prob > chi2       =     0.0000
Log pseudolikelihood = -131.53859               Pseudo R2         =     0.0981

------------------------------------------------------------------------------
             |               Robust
     death2w |        IRR   Std. Err.      z    P>|z|     [95% Conf. Interval]
-------------+----------------------------------------------------------------
  IMCI_refer |
        yes  |   3.053294   1.433007     2.38   0.017      1.21695    7.660629
             |
     hypoxia |
      90-93  |   4.234005   2.181047     2.80   0.005     1.542677    11.62058
        <90  |   10.19301   5.958925     3.97   0.000     3.241023    32.05697
     failed  |   10.88155   7.174519     3.62   0.000     2.988609    39.61981
             |
    distance |   1.029863   .0266113     1.14   0.255     .9790045    1.083363
```

```
       _cons |   .0022019   .0009653   -13.96   0.000     .0009325    .0051994
------------------------------------------------------------------------------
Note: _cons estimates baseline incidence rate.

Measures of Fit for poisson of death2w
```

	Model B	Model A	Difference
Model:	poisson	poisson	
N:	3848	3848	0
BIC:	-31437.309	-31444.231	6.922

Difference of 6.922 in BIC provides strong support for <u>Model A</u>.

- **PCV (pneumococcal conjugate vaccine) status**

<u>Model A:</u>

```
Poisson regression                              Number of obs     =      3,848
                                                Wald chi2(4)      =      45.28
                                                Prob > chi2       =     0.0000
Log pseudolikelihood = -132.20513               Pseudo R2         =     0.0936

------------------------------------------------------------------------------
             |               Robust
     death2w |        IRR   Std. Err.      z    P>|z|     [95% Conf. Interval]
-------------+----------------------------------------------------------------
  IMCI_refer|
        yes  |   3.088078   1.488237     2.34   0.019     1.200794    7.941595
             |
     hypoxia |
      90-93  |   4.297255   2.224147     2.82   0.005      1.55823    11.85088
        <90  |   10.25691   6.015159     3.97   0.000     3.249602    32.37451
     failed  |   11.39268   7.505195     3.69   0.000       3.1324    41.43571
             |
       _cons |   .0030693   .0009125   -19.46   0.000     .0017139    .0054966
------------------------------------------------------------------------------
Note: _cons estimates baseline incidence rate.
```

<u>Model B:</u>

```
Poisson regression                              Number of obs     =      3,848
                                                Wald chi2(6)      =      60.70
                                                Prob > chi2       =     0.0000
Log pseudolikelihood = -128.17505               Pseudo R2         =     0.1212

------------------------------------------------------------------------------
             |               Robust
     death2w |        IRR   Std. Err.      z    P>|z|     [95% Conf. Interval]
-------------+----------------------------------------------------------------
  IMCI_refer|
        yes  |   3.103873   1.473752     2.39   0.017     1.223896    7.871608
             |
     hypoxia |
      90-93  |   4.338794   2.252709     2.83   0.005     1.568281    12.00368
        <90  |   11.94653   7.113074     4.17   0.000     3.719022    38.37559
     failed  |    8.65485   5.661911     3.30   0.001     2.401113    31.19655
             |
         PCV|
        >=1  |   1.103826   1.191732     0.09   0.927     .1330184    9.159875
       miss  |   3.627252   3.864158     1.21   0.226     .4495539    29.26669
             |
       _cons |   .0017436   .0018988    -5.83   0.000     .0002063    .0147371
------------------------------------------------------------------------------
Note: _cons estimates baseline incidence rate.
```

	Model B	Model A	Difference
Model:	poisson	poisson	
N:	3848	3848	0
BIC:	-31427.525	-31444.231	16.706

Difference of 16.706 in BIC provides **<u>very strong support</u>** for **Model A**.

- **Maternal age in years (continuous)**

Model A:

```
Poisson regression                              Number of obs     =      3,848
                                                Wald chi2(4)      =      45.28
                                                Prob > chi2       =     0.0000
Log pseudolikelihood = -132.20513               Pseudo R2         =     0.0936

------------------------------------------------------------------------------
             |               Robust
     death2w |        IRR   Std. Err.      z    P>|z|     [95% Conf. Interval]
-------------+----------------------------------------------------------------
  IMCI_refer |
        yes  |   3.088078   1.488237     2.34   0.019     1.200794    7.941595
             |
     hypoxia |
      90-93  |   4.297255   2.224147     2.82   0.005      1.55823    11.85088
        <90  |   10.25691   6.015159     3.97   0.000     3.249602    32.37451
     failed  |   11.39268   7.505195     3.69   0.000       3.1324    41.43571
             |
       _cons |   .0030693   .0009125   -19.46   0.000     .0017139    .0054966
------------------------------------------------------------------------------
Note: _cons estimates baseline incidence rate.
```

Model B:

```
Poisson regression                              Number of obs     =      3,530
                                                Wald chi2(5)      =      64.19
                                                Prob > chi2       =     0.0000
Log pseudolikelihood = -128.75755               Pseudo R2         =     0.1045

------------------------------------------------------------------------------
             |               Robust
     death2w |        IRR   Std. Err.      z    P>|z|     [95% Conf. Interval]
-------------+----------------------------------------------------------------
  IMCI_refer |
        yes  |   3.172813   1.509903     2.43   0.015     1.248442    8.063449
             |
     hypoxia |
      90-93  |   4.242387   2.203014     2.78   0.005     1.533178    11.73891
        <90  |   11.05542   6.275122     4.23   0.000      3.63436    33.62965
     failed  |   11.00372   7.213225     3.66   0.000     3.044774    39.76713
             |
  mother_age |   1.046184   .0309808     1.52   0.127     .9871911    1.108702
       _cons |   .0009017   .0007646    -8.27   0.000     .0001711    .0047516
------------------------------------------------------------------------------
Note: _cons estimates baseline incidence rate.

Measures of Fit for poisson of death2w
```

	Model B	Model A	Difference
Model:	poisson	poisson	
N:	3530	3848	-318
BIC:	-28513.890	-31444.231	2930.341

Difference of 2930.341 in BIC provides strong support for Model A.

- **Wealth quintile (socioeconomic status)**

Model A:

```
Poisson regression                              Number of obs     =      3,848
                                                Wald chi2(4)      =      45.28
                                                Prob > chi2       =     0.0000
Log pseudolikelihood = -132.20513               Pseudo R2         =     0.0936

------------------------------------------------------------------------------
             |               Robust
     death2w |        IRR   Std. Err.      z    P>|z|     [95% Conf. Interval]
-------------+----------------------------------------------------------------
  IMCI_refer |
        yes  |   3.088078   1.488237     2.34   0.019     1.200794    7.941595
```

```
             |
     hypoxia |
      90-93  |   4.297255   2.224147     2.82   0.005      1.55823    11.85088
        <90  |   10.25691   6.015159     3.97   0.000     3.249602    32.37451
     failed  |   11.39268   7.505195     3.69   0.000       3.1324    41.43571
             |
       _cons |   .0030693   .0009125   -19.46   0.000     .0017139    .0054966
------------------------------------------------------------------------------
Note: _cons estimates baseline incidence rate.
```

Model B:

```
Poisson regression                              Number of obs     =      3,557
                                                Wald chi2(5)      =      38.94
                                                Prob > chi2       =     0.0000
Log pseudolikelihood =  -126.5535               Pseudo R2         =     0.0892

------------------------------------------------------------------------------
             |               Robust
     death2w |        IRR   Std. Err.      z    P>|z|     [95% Conf. Interval]
-------------+----------------------------------------------------------------
 IMCI_refer  |
        yes  |   3.251311   1.588674     2.41   0.016     1.247784    8.471835
             |
     hypoxia |
      90-93  |   4.344434    2.24479     2.84   0.004     1.578023     11.9606
        <90  |   7.701106   5.007334     3.14   0.002     2.153246    27.54309
     failed  |   11.89159   7.765656     3.79   0.000     3.306539    42.76678
             |
 ses_assetgr |   .9314847    .134677    -0.49   0.623     .7016271    1.236645
       _cons |   .0040145   .0019706   -11.24   0.000     .0015339    .0105065
------------------------------------------------------------------------------
Note: _cons estimates baseline incidence rate.

Measures of Fit for poisson of death2w
```

	Model B	Model A	Difference
Model:	poisson	poisson	
N:	3557	3848	-291
BIC:	-28765.905	-31444.231	2678.326

Difference of 2678.326 in BIC provides strong support for Model A.

- **Cooking source (indoor air pollution)**

Model A:

```
Poisson regression                              Number of obs     =      3,848
                                                Wald chi2(4)      =      45.28
                                                Prob > chi2       =     0.0000
Log pseudolikelihood = -132.20513               Pseudo R2         =     0.0936

------------------------------------------------------------------------------
             |               Robust
     death2w |        IRR   Std. Err.      z    P>|z|     [95% Conf. Interval]
-------------+----------------------------------------------------------------
 IMCI_refer  |
        yes  |   3.088078   1.488237     2.34   0.019     1.200794    7.941595
             |
     hypoxia |
      90-93  |   4.297255   2.224147     2.82   0.005      1.55823    11.85088
        <90  |   10.25691   6.015159     3.97   0.000     3.249602    32.37451
     failed  |   11.39268   7.505195     3.69   0.000       3.1324    41.43571
             |
       _cons |   .0030693   .0009125   -19.46   0.000     .0017139    .0054966
------------------------------------------------------------------------------
Note: _cons estimates baseline incidence rate.
```

Model B:

```
Poisson regression                              Number of obs     =      3,550
                                                Wald chi2(5)      =      40.69
                                                Prob > chi2       =     0.0000
Log pseudolikelihood = -126.58884               Pseudo R2         =     0.0886
```

death2w	IRR	Robust Std. Err.	z	P>|z|	[95% Conf. Interval]	
IMCI_refer						
yes	3.33858	1.591857	2.53	0.011	1.311303	8.500029
hypoxia						
90-93	4.32805	2.23504	2.84	0.005	1.572986	11.90857
<90	7.759166	5.074746	3.13	0.002	2.153285	27.95944
failed	11.92115	7.77758	3.80	0.000	3.318777	42.82111
cook	1.096524	1.128305	0.09	0.929	.1459303	8.2393
_cons	.002981	.003186	-5.44	0.000	.000367	.0242163

Note: _cons estimates baseline incidence rate.

Measures of Fit for poisson of death2w

	Model B	Model A	Difference
Model:	poisson	poisson	
N:	3550	3848	-298
BIC:	-28701.620	-31444.231	2742.611

Difference of 2742.611 in BIC provides strong support for Model A.

- **Water source**

Model A:

```
Poisson regression                              Number of obs     =      3,848
                                                Wald chi2(4)      =      45.28
                                                Prob > chi2       =     0.0000
Log pseudolikelihood = -132.20513               Pseudo R2         =     0.0936
```

death2w	IRR	Robust Std. Err.	z	P>|z|	[95% Conf. Interval]	
IMCI_refer						
yes	3.088078	1.488237	2.34	0.019	1.200794	7.941595
hypoxia						
90-93	4.297255	2.224147	2.82	0.005	1.55823	11.85088
<90	10.25691	6.015159	3.97	0.000	3.249602	32.37451
failed	11.39268	7.505195	3.69	0.000	3.1324	41.43571
_cons	.0030693	.0009125	-19.46	0.000	.0017139	.0054966

Note: _cons estimates baseline incidence rate.

Model B:

```
Poisson regression                              Number of obs     =      3,558
                                                Wald chi2(5)      =      39.50
                                                Prob > chi2       =     0.0000
Log pseudolikelihood = -125.79688               Pseudo R2         =     0.0947
```

death2w	IRR	Robust Std. Err.	z	P>|z|	[95% Conf. Interval]	
IMCI_refer						
yes	3.420568	1.648687	2.55	0.011	1.329921	8.79773
hypoxia						
90-93	3.988581	2.099558	2.63	0.009	1.421519	11.1914
<90	7.26988	4.774762	3.02	0.003	2.006639	26.33815
failed	11.34992	7.435438	3.71	0.000	3.143133	40.9848
water	.5385543	.236354	-1.41	0.158	.2278582	1.2729
_cons	.0071539	.0043359	-8.15	0.000	.0021809	.0234666

```
------------------------------------------------------------------------------
Note: _cons estimates baseline incidence rate.

Measures of Fit for poisson of death2w
```

	Model B	Model A	Difference
Model:	poisson	poisson	
N:	3558	3848	-290
BIC:	-28776.592	-31444.231	2667.638

Difference of 2667.638 in BIC provides strong support for Model A.

- **Type of toilet (sanitation)**

Model A:

```
Poisson regression                              Number of obs     =      3,848
                                                Wald chi2(4)      =      45.28
                                                Prob > chi2       =     0.0000
Log pseudolikelihood = -132.20513               Pseudo R2         =     0.0936

------------------------------------------------------------------------------
             |               Robust
     death2w |        IRR   Std. Err.      z    P>|z|     [95% Conf. Interval]
-------------+----------------------------------------------------------------
  IMCI_refer |
        yes  |   3.088078   1.488237     2.34   0.019     1.200794    7.941595
             |
     hypoxia |
      90-93  |   4.297255   2.224147     2.82   0.005      1.55823    11.85088
        <90  |   10.25691   6.015159     3.97   0.000     3.249602    32.37451
     failed  |   11.39268   7.505195     3.69   0.000       3.1324    41.43571
             |
       _cons |   .0030693   .0009125   -19.46   0.000     .0017139    .0054966
------------------------------------------------------------------------------
Note: _cons estimates baseline incidence rate.
```

Model B:

```
Poisson regression                              Number of obs     =      3,556
                                                Wald chi2(5)      =      39.09
                                                Prob > chi2       =     0.0000
Log pseudolikelihood = -126.57305               Pseudo R2         =     0.0890

------------------------------------------------------------------------------
             |               Robust
     death2w |        IRR   Std. Err.      z    P>|z|     [95% Conf. Interval]
-------------+----------------------------------------------------------------
  IMCI_refer |
        yes  |   3.377691   1.606776     2.56   0.011     1.329539    8.581016
             |
     hypoxia |
      90-93  |   4.343734   2.245523     2.84   0.004      1.57699    11.96459
        <90  |   7.998167    5.19565     3.20   0.001     2.238956    28.57165
     failed  |   11.79332   7.674036     3.79   0.000     3.294201    42.22035
             |
      toilet |   1.049474   .3975409     0.13   0.899     .4995008    2.204992
       _cons |   .0031434   .0012193   -14.86   0.000     .0014697    .0067233
------------------------------------------------------------------------------
Note: _cons estimates baseline incidence rate.

Measures of Fit for poisson of death2w
```

	Model B	Model A.	Difference
Model:	poisson	poisson	
N:	3556	3848	-292
BIC:	-28756.691	-31444.231	2687.540

Difference of 2687.540 in BIC provides strong support for Model A.

◊ **Exploratory post-hoc sensitivity analysis – Weekly community surveillance for acute respiratory illness**

- **Supplemental Table 16. Hypoxemia strata**

SpO_2 range, n (%)	No weekly surveillance, N=3,340	Weekly surveillance, N=508	p value
**94-100%**	2,932 (87.7%)	441 (86.8%)	0.730
**90-93%**	261 (7.8%)	45 (8.8%)	
**<90%**	87 (2.6%)	15 (2.9%)	
**Failed measurement**	60 (1.8%)	7 (1.3%)	

- **Supplemental Table 17. Mortality**

Mortality, n (%)	No weekly surveillance, N=3,340	Weekly surveillance, N=508	p value
**Alive**	3,317 (99.3%)	507 (99.8%)	0.190
**Dead**	23 (0.7%)	1 (0.2%)	

- **Primary Model controlling for weekly surveillance (Stata output)**

```
Poisson regression                              Number of obs     =      3,848
                                                Wald chi2(5)      =      48.46
                                                Prob > chi2       =     0.0000
Log pseudolikelihood = -130.91839               Pseudo R2         =     0.1024

--------------------------------------------------------------------------------
                |               Robust
        death2w |        IRR   Std. Err.      z    P>|z|     [95% Conf. Interval]
----------------+---------------------------------------------------------------
     IMCI_refer |
            yes |   3.201104   1.506542     2.47   0.013     1.272615    8.051975
                |
           SpO₂ |
          90-93 |   4.280017   2.208068     2.82   0.005     1.557074    11.76473
            <90 |   10.50013   6.142024     4.02   0.000     3.336463    33.04478
         failed |   11.29997   7.392041     3.71   0.000     3.135101    40.72895
                |
         Weekly |
     yes Weekly |   .2656409   .2692679    -1.31   0.191      .036431    1.936951
          _cons |   .0033847   .0010095   -19.07   0.000     .0018865    .0060729
--------------------------------------------------------------------------------
Note: _cons estimates baseline incidence rate.
```

- **Model selection accounting for weekly surveillance (Stata output)**

Model A.

```
Poisson regression                              Number of obs     =      3,848
                                                Wald chi2(4)      =      45.28
                                                Prob > chi2       =     0.0000
Log pseudolikelihood = -132.20513               Pseudo R2         =     0.0936

-----------------------------------------------------------------------------
             |               Robust
     death2w |        IRR   Std. Err.      z    P>|z|     [95% Conf. Interval]
-------------+---------------------------------------------------------------
  IMCI_refer |
         yes |   3.088078   1.488237     2.34   0.019     1.200794    7.941595
             |
        SpO₂ |
       90-93 |   4.297255   2.224147     2.82   0.005      1.55823    11.85088
         <90 |   10.25691   6.015159     3.97   0.000     3.249602    32.37451
      failed |   11.39268   7.505195     3.69   0.000       3.1324    41.43571
             |
       _cons |   .0030693   .0009125   -19.46   0.000     .0017139    .0054966
-----------------------------------------------------------------------------
Note: _cons estimates baseline incidence rate.
```

Model B.

```
Poisson regression                              Number of obs     =      3,848
                                                Wald chi2(5)      =      48.46
                                                Prob > chi2       =     0.0000
Log pseudolikelihood = -130.91839               Pseudo R2         =     0.1024

--------------------------------------------------------------------------------
               |               Robust
       death2w |        IRR   Std. Err.      z    P>|z|     [95% Conf. Interval]
---------------+----------------------------------------------------------------
    IMCI_refer |
          yes  |   3.201104   1.506542     2.47   0.013     1.272615    8.051975
               |
          SpO2 |
        90-93  |   4.280017   2.208068     2.82   0.005     1.557074    11.76473
          <90  |   10.50013   6.142024     4.02   0.000     3.336463    33.04478
       failed  |   11.29997   7.392041     3.71   0.000     3.135101    40.72895
               |
        Weekly |
    yes Weekly |   .2656409   .2692679    -1.31   0.191      .036431    1.936951
         _cons |   .0033847   .0010095   -19.07   0.000     .0018865    .0060729
--------------------------------------------------------------------------------
Note: _cons estimates baseline incidence rate.

                                      Model B          Model A        Difference
Model:                                poisson          poisson
N:                                       3848             3848             0
BIC:                               -31430.294       -31444.231          13.937
```

Difference of 13.937 in BIC provides very strong support for Model A.

- **Subgroup analysis of Primary Model dropping 508 weekly surveillance cases (Stata output)**

```
Poisson regression                              Number of obs     =      3,340
                                                Wald chi2(4)      =      39.59
                                                Prob > chi2       =     0.0000
Log pseudolikelihood = -123.97964               Pseudo R2         =     0.0983

-----------------------------------------------------------------------------
            |               Robust
    death2w |        IRR   Std. Err.      z    P>|z|     [95% Conf. Interval]
------------+----------------------------------------------------------------
 IMCI_refer |
       yes  |   2.674457   1.341413     1.96   0.050     1.000698    7.147733
            |
       SpO2 |
     90-93  |   4.754256   2.470648     3.00   0.003     1.716872    13.16519
       <90  |   11.72219    6.85962     4.21   0.000     3.723063     36.9077
    failed  |   12.51183   8.190208     3.86   0.000     3.468383    45.13515
            |
      _cons |   .0033431    .001044   -18.26   0.000     .0018127    .0061655
-----------------------------------------------------------------------------
Note: _cons estimates baseline incidence rate.
```

◊ Exploratory post-hoc sensitivity analysis – One month mortality

24 of the 33 deaths (73%) that took place by four-weeks had already occurred by the two-week mark. To evaluate whether using a four-week endpoint meaningfully changed our findings we conducted a post-hoc analysis of our primary model using a four-week outcome period. As is seen below there are no qualitative changes in our findings based on this sensitivity analysis.

```
Poisson regression                              Number of obs     =      3,846
                                                Wald chi2(4)      =      41.97
                                                Prob > chi2       =     0.0000
Log pseudolikelihood = -177.01457               Pseudo R2         =     0.0685

------------------------------------------------------------------------------
             |               Robust
     death1m |        IRR   Std. Err.      z    P>|z|     [95% Conf. Interval]
-------------+----------------------------------------------------------------
  IMCI refer |
        yes  |   3.091602   1.281484     2.72   0.006     1.372008    6.966435
             |
        SpO₂ |
      90-93  |   3.797379   1.649481     3.07   0.002     1.620856    8.896584
        <90  |   6.474083   3.604245     3.36   0.001     2.174193    19.27784
     failed  |   7.190706   4.537666     3.13   0.002     2.087489    24.76959
             |
       _cons |   .0048611   .0011449   -22.62   0.000     .0030638    .0077128
------------------------------------------------------------------------------
Note: _cons estimates baseline incidence rate.
```

◊ **Exploratory post-hoc sensitivity analysis – Verbal autopsy data**

Surveillance activities include verbal autopsies on deceased individuals conducted by trained CHWs using a standardized form adapted from the WHO. Post-hoc sensitivity analyses of verbal autopsy data were performed using two approaches, (1) a "symptom coding" approach aligned with IMCI and (2) a computer coded verbal autopsy (CCVA) approach using with WHO's OpenVA platform (https://openva.net/).

IMCI symptom coding:

In the symptom coding VA analysis approach, we created a binary variable for probable LRI using two closed-ended questions from the WHO VA tool, (1) "Did baby/child have any difficulty with breathing?" and (2) "Did baby/child have cough?" We then classified a child with cough and/or difficult breathing and either fast breathing, chest indrawing, grunting, or wheezing as a probable LRI death.

2 week mortality:

Using this approach, VA data indicates that at our primary 2 week outcome, 21/24 deaths were probable LRI (87%). We re-analyzed our primary model dropping the 3 non probable LRI deaths at two weeks from our analysis (1 death occurred in the SpO_2 94-100% range, 1 death in <90%, and 1 death in failed SpO_2 measurement). There are no qualitative changes in our findings based on this sensitivity analysis.

Supplemental Table 18. IMCI symptom VA results by probable LRI and SpO_2 strata among 3-11 month olds – 2 week mortality

**Hypoxemia, n (%)**	**IMCI symptom VA – No LRI, N=3**	**IMCI symptom VA – LRI, N=21**
**SpO_2 94-100%**	1 (8%)	11 (91%)
**SpO_2 90-93%**	0	5 (100%)
**SpO_2 <90%**	1 (25%)	3 (75%)
**Failed SpO_2 measurement**	1 (33%)	2 (66%)

Statistical Output (Stata). Risk of two-week LRI mortality by IMCI symptom VA results among 3-11 month olds

```
Poisson regression                              Number of obs     =      3,845
                                                Wald chi2(4)      =      35.95
                                                Prob > chi2       =     0.0000
Log pseudolikelihood = -119.10737               Pseudo R2         =     0.0867

------------------------------------------------------------------------------
             |               Robust
     death14 |        IRR   Std. Err.      z    P>|z|     [95% Conf. Interval]
-------------+----------------------------------------------------------------
  IMCI refer |
         yes |   3.766143   1.850566     2.70   0.007     1.437615    9.866223
             |
       SpO2  |
       90-93 |   4.607234   2.415904     2.91   0.004     1.648519    12.87616
         <90 |   8.297839   5.516088     3.18   0.001     2.254855    30.53595
      failed |   8.167038   6.428539     2.67   0.008     1.746049    38.20083
             |
       _cons |   .0026942   .0008531   -18.69   0.000     .0014484    .0050113
------------------------------------------------------------------------------
Note: _cons estimates baseline incidence rate.
```

1 month mortality:

At 1 month, 29/33 (87%) deaths were "probable LRI" using the IMCI symptom coding approach. We re-analyzed our primary model dropping the 4 non probable LRI deaths at 3 months from our analysis (2 deaths occurred in the SpO_2 94-100% range, 0 deaths 90-93% range, 1 death in <90%, and 1 death in failed SpO_2 measurement). We found no qualitative changes in our findings with this sensitivity analysis.

Supplemental Table 19. WHO IMCI symptom VA results by probable LRI and SpO_2 strata among 3-11 month olds – 1 month mortality

**Hypoxemia, n (%)**	**WHO IMCI symptom VA – No LRI, N=4**	**WHO IMCI symptom VA – LRI, N=29**
**SpO_2 94-100%**	2 (10%)	17 (89%)
**SpO_2 90-93%**	0	7 (100%)
**SpO_2 <90%**	1 (25%)	3 (75%)
**Failed SpO_2 measurement**	1 (33%)	2 (66%)

Statistical Output (Stata). Risk of 1-month LRI mortality by WHO IMCI symptom VA results among 3-11 month olds

```
Poisson regression                               Number of obs     =      3,842
                                                 Wald chi2(4)      =      37.00
                                                 Prob > chi2       =     0.0000
Log pseudolikelihood = -158.96458                Pseudo R2         =     0.0688

------------------------------------------------------------------------------
             |               Robust
     death1m |        IRR   Std. Err.      z    P>|z|     [95% Conf. Interval]
-------------+----------------------------------------------------------------
 IMCI_refer |
        yes  |   3.714124   1.571479     3.10   0.002      1.62071    8.511526
             |
     hypoxia |
      90-93  |   4.175754   1.847313     3.23   0.001     1.754573    9.937984
        <90  |   5.372659   3.420464     2.64   0.008     1.542688    18.71115
     failed  |   5.290618   4.030701     2.19   0.029     1.188534    23.55057
             |
       _cons |   .0041805   .0010546   -21.71   0.000     .0025497    .0068542
------------------------------------------------------------------------------
Note: _cons estimates baseline incidence rate.
```

3 month mortality:

At 3 months, 39/47 (83%) deaths were "probable LRI" using the IMCI symptom coding approach. We re-analyzed our primary model dropping the 8 non probable LRI deaths at 3 months from our analysis (5 deaths occurred in the SpO_2 94-100% range, 1 death 90-93% range, 1 death in <90%, and 1 death in failed SpO_2 measurement). Similarly, we found no qualitative changes in our findings with this sensitivity analysis.

Supplemental Table 20. IMCI symptom VA results by probable LRI and SpO_2 strata among 3-11 month olds – 3 month mortality

Hypoxemia, n (%)	IMCI symptom VA – No LRI, N=8	IMCI symptom VA – LRI, N=39
**SpO_2 94-100%**	5 (17%)	24 (82%)
**SpO_2 90-93%**	1 (11%)	8 (88%)
**SpO_2 <90%**	1 (25%)	3 (75%)
**Failed SpO_2 measurement**	1 (33%)	4 (80%)

Statistical Output (Stata). Risk of 3 month LRI mortality by IMCI symptom VA results among 3-11 month olds

```
Poisson regression                               Number of obs     =      3,829
                                                 Wald chi2(4)      =      49.76
                                                 Prob > chi2       =     0.0000
Log pseudolikelihood = -201.32078                Pseudo R2         =     0.0760

------------------------------------------------------------------------------
             |               Robust
     Death3m |        IRR   Std. Err.      z    P>|z|     [95% Conf. Interval]
-------------+----------------------------------------------------------------
 IMCI refer  |
        yes  |   4.398712   1.512742     4.31   0.000     2.241778    8.630948
             |
        SpO2 |
      90-93  |   3.359161   1.352153     3.01   0.003     1.526157    7.393709
        <90  |   3.727549   2.319262     2.11   0.034     1.101057    12.61934
     failed  |   7.295683   3.735095     3.88   0.000     2.674762    19.89971
             |
       _cons |   .0056763   .0012467   -23.55   0.000     .0036908    .0087301
------------------------------------------------------------------------------
Note: _cons estimates baseline incidence rate.
```

CCVA approach:

For the CCVA approach we used the WHO's OpenVA software to generate a binary variable of probable LRI death if the primary cause of death was due to LRI and coded as any of "01.02 Acute Respiratory infection, including pneumonia," "01.06 Measles," or "01.10 Pertussis." Using this approach, VA data indicates that 20/23 deaths (86%) by two weeks and 36/45 deaths (80%) by 3 months were "probable LRI." (1 death by 2 weeks and 2 deaths by 3 months had insufficient data to be processed using Open VA software program).

2 week mortality:

We re-analyzed our primary model dropping the 3 non probable LRI deaths at two weeks from our analysis (1 non LRI death occurred in the SpO_2 94-100% range, 1 death in the 90-93% range, 0 deaths in <90%, and 1 death in failed SpO_2 measurement). There are no qualitative changes in our findings based on this sensitivity analysis.

Supplemental Table 21. CCVA results by probable LRI and SpO_2 strata among 3-11 month olds – 2 week mortality

**Hypoxemia, n (%)**	**CCVA – No LRI, N=3**	**CCVA – LRI, N=20**
**SpO_2 94-100%**	1 (9%)	10 (90%)
**SpO_2 90-93%**	1 (20%)	4 (80%)
**SpO_2 <90%**	0	4 (100%)
**Failed SpO_2 measurement**	1 (33%)	2 (66%)

Statistical Output (Stata). Risk of two-week LRI mortality by CCVA results among 3-11 month olds

```
Poisson regression                              Number of obs     =      3,838
                                                Wald chi2(4)      =      44.90
                                                Prob > chi2       =     0.0000
Log pseudolikelihood = -118.12924               Pseudo R2         =     0.0939

------------------------------------------------------------------------------
             |               Robust
     death2w |        IRR   Std. Err.      z    P>|z|     [95% Conf. Interval]
-------------+----------------------------------------------------------------
  IMCI_refer |
        yes  |   3.837677   1.884962     2.74   0.006     1.465485    10.04975
             |
     hypoxia |
      90-93  |   3.727059   2.097485     2.34   0.019     1.236898    11.23049
        <90  |   10.94352   6.547705     4.00   0.000     3.387411    35.35463
     failed  |   8.488399    6.63871     2.73   0.006     1.832769    39.31369
             |
       _cons |   .0026866   .0008533   -18.64   0.000     .0014416    .0050066
------------------------------------------------------------------------------
Note: _cons estimates baseline incidence rate.
```

1 month mortality:

We re-analyzed our primary model dropping the 3 non probable LRI deaths at 1 month from our analysis (2 non LRI deaths occurred in the SpO_2 94-100% range, 2 deaths in the 90-93% range, 0 deaths in <90%, and 1 death in failed SpO_2 measurement). There are no qualitative changes in our findings based on this sensitivity analysis.

Supplemental Table 22. CCVA results by probable LRI and SpO_2 strata among 3-11 month olds – 1 month mortality

**Hypoxemia, n (%)**	**CCVA – No LRI, N=5**	**CCVA – LRI, N=27**
**SpO_2 94-100%**	2 (11%)	16 (88%)
**SpO_2 90-93%**	2 (28%)	5 (71%)
**SpO_2 <90%**	0	4 (100%)
**Failed SpO_2 measurement**	1 (33%)	2 (84%)

Statistical Output (Stata). Risk of 1 month LRI mortality by CCVA results among 3-11 month olds

```
Poisson regression                              Number of obs     =      3,836
                                                Wald chi2(4)      =      41.41
                                                Prob > chi2       =     0.0000
Log pseudolikelihood = -154.03109               Pseudo R2         =     0.0708

------------------------------------------------------------------------------
             |               Robust
     Death1m |        IRR   Std. Err.      z    P>|z|     [95% Conf. Interval]
-------------+----------------------------------------------------------------
   IMCI_refer |
        yes  |   3.999751   1.701045     3.26   0.001     1.737911    9.205311
             |
```

```
     hypoxia |
       90-93  |   3.002195   1.485697     2.22   0.026     1.138163    7.919053
         <90  |   7.048637   4.005814     3.44   0.001     2.313974    21.47098
      failed  |   5.464678   4.148496     2.24   0.025     1.234176    24.19647
              |
       _cons |   .0041123    .001054   -21.44   0.000     .0024884    .0067958
------------------------------------------------------------------------------
Note: _cons estimates baseline incidence rate.
```

3 month mortality:

We re-analyzed our primary model dropping the 3 non probable LRI deaths at 3 months from our analysis (5 non LRI deaths occurred in the SpO_2 94-100% range, 3 deaths in the 90-93% range, 0 deaths in <90%, and 1 death in failed SpO_2 measurement). There are no qualitative changes in our findings based on this sensitivity analysis.

Supplemental Table 23. CCVA results by probable LRI and SpO_2 strata among 3-11 month olds – 3 month mortality

**Hypoxemia, n (%)**	**CCVA – No LRI, N=9**	**CCVA – LRI, N=36**
**SpO_2 94-100%**	5 (18%)	22 (81%)
**SpO_2 90-93%**	3 (33%)	6 (66%)
**SpO_2 <90%**	0	4 (100%)
**Failed SpO_2 measurement**	1 (20%)	4 (80%)

Statistical Output (Stata). Risk of 3 month LRI mortality by CCVA results among 3-11 month olds

```
Poisson regression                                Number of obs     =      3,827
                                                  Wald chi2(4)      =      44.22
                                                  Prob > chi2       =     0.0000
Log pseudolikelihood = -194.85037                 Pseudo R2         =     0.0661

------------------------------------------------------------------------------
             |               Robust
     Death3m |        IRR   Std. Err.      z    P>|z|     [95% Conf. Interval]
-------------+----------------------------------------------------------------
  IMCI_refer |
         yes  |   4.305268    1.55199     4.05   0.000     2.123996    8.726633
              |
     hypoxia |
       90-93  |   2.536414    1.13168     2.09   0.037     1.057881      6.0814
         <90  |   4.946178   2.724373     2.90   0.004     1.680447    14.55843
      failed  |   5.747006   3.435861     2.92   0.003     1.780528     18.5496
              |
       _cons |   .0057086   .0012562   -23.48   0.000     .0037087     .008787
------------------------------------------------------------------------------
Note: _cons estimates baseline incidence rate.
```

◊ **Exploratory post-hoc sensitivity analysis – Pneumococcal conjugate vaccine (PCV) data**

Supplemental Table 24. PCV status and death by two weeks

PCV status	Alive N=3,824	Dead N=24	Total N=3,848	p value
**Zero PCV doses, n (%)**	189 (4.9%)	1 (4.1%)	190 (4.9%)	0.008
**>1 PCV dose, n (%)**	2,634 (68.8%)	10 (41.6%)	2,644 (68.7%)	
**Missing, n (%)**	1,001 (26.1%)	13 (54.1%)	1,014 (26.3%)	

Primary model controlling for participant PCV status

```
Poisson regression                              Number of obs     =      3,848
                                                Wald chi2(6)      =      60.70
                                                Prob > chi2       =     0.0000
Log pseudolikelihood = -128.17505               Pseudo R2         =     0.1212

------------------------------------------------------------------------------
             |               Robust
     death2w |        IRR   Std. Err.      z    P>|z|     [95% Conf. Interval]
-------------+----------------------------------------------------------------
   IMCI_refer|
         yes |   3.103873   1.473752     2.39   0.017     1.223896    7.871608
             |
     hypoxia |
       90-93 |   4.338794   2.252709     2.83   0.005     1.568281    12.00368
         <90 |   11.94653   7.113074     4.17   0.000     3.719022    38.37559
      failed |    8.65485   5.661911     3.30   0.001     2.401113    31.19655
             |
          PCV|
         >=1 |   1.103826   1.191732     0.09   0.927     .1330184    9.159875
        miss |   3.627252   3.864158     1.21   0.226     .4495539    29.26669
             |
       _cons |   .0017436   .0018988    -5.83   0.000     .0002063    .0147371
------------------------------------------------------------------------------
Note: _cons estimates baseline incidence rate.
```

Subgroup analysis of participants with a *known* PCV status (drops 1,014 participants and 13/24 mortality events)

```
Poisson regression                              Number of obs     =      2,834
                                                Wald chi2(4)      =    2860.19
                                                Prob > chi2       =     0.0000
Log pseudolikelihood = -63.776412               Pseudo R2         =     0.1150

------------------------------------------------------------------------------
             |               Robust
     death2w |        IRR   Std. Err.      z    P>|z|     [95% Conf. Interval]
-------------+----------------------------------------------------------------
  IMCI_refer |
         yes |   5.383291   3.621734     2.50   0.012     1.440066    20.12396
             |
     hypoxia |
       90-93 |   3.295145   2.679415     1.47   0.143     .6694655    16.21888
         <90 |   12.70178   9.671931     3.34   0.001     2.855659    56.49666
      failed |   4.41e-09   2.13e-09   -39.80   0.000     1.71e-09    1.14e-08
             |
       _cons |      .0018   .0007297   -15.59   0.000     .0008132    .0039844
------------------------------------------------------------------------------
Note: _cons estimates baseline incidence rate.
```

◊ **Exploratory post-hoc sensitivity analysis – aRR by SpO_2 unit (1%) using the final model at two-week mortality among 3-11 month olds with suspected pneumonia**

```
Poisson regression                               Number of obs     =      3,781
                                                 Wald chi2(2)      =      45.29
                                                 Prob > chi2       =     0.0000
Log pseudolikelihood = -121.26393                Pseudo R2         =     0.0676

------------------------------------------------------------------------------
             |               Robust
     death14 |        IRR   Std. Err.      z    P>|z|     [95% Conf. Interval]
-------------+----------------------------------------------------------------
IMCI referral |
         yes  |   4.026634   2.014084     2.78   0.005     1.510704     10.7326
        SpO₂ |  .9157032   .0156097    -5.17   0.000     .8856143    .9468145
       _cons |   19.68109   32.70737     1.79   0.073     .7576438      511.25
------------------------------------------------------------------------------
Note: _cons estimates baseline incidence rate.
```

◊ **Supplemental Figure 2. Exploratory post-hoc sensitivity analysis – probability of mortality by SpO_2 1% units between 90-100% among 3-11 month olds with suspected pneumonia**

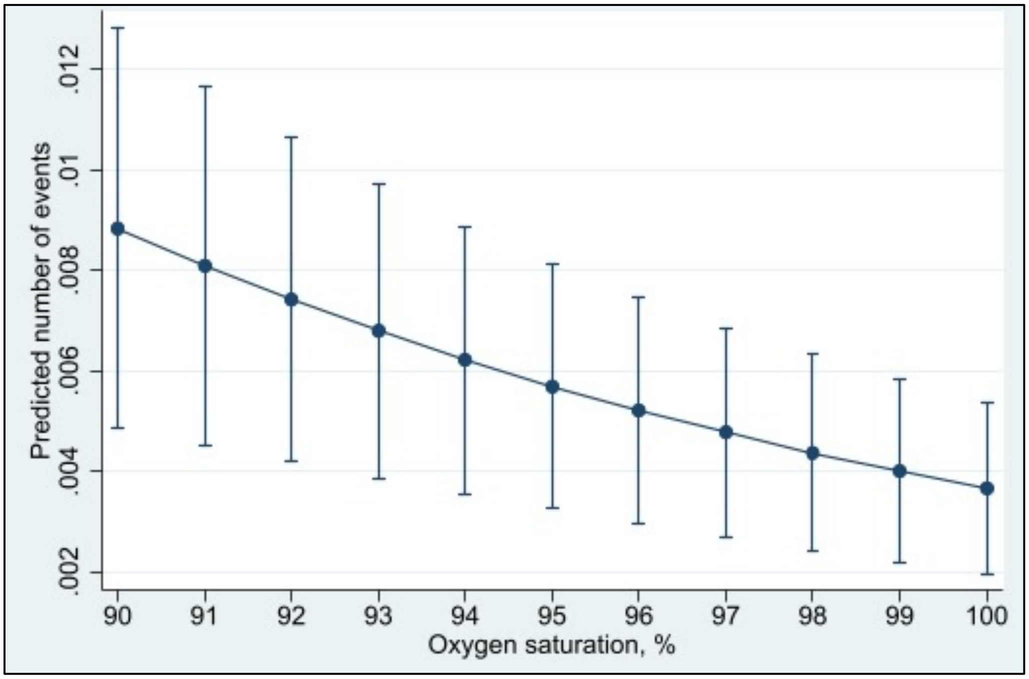

Supplement: Supplementary appendix [file mmc1.pdf]
